# Five-Biomarker Definition for Hypervirulent *Klebsiella pneumoniae* Bloodstream Infection and Abscess Complications, Japan, 2022–2024

**DOI:** 10.3201/eid3211.260825

**Published:** 2026-11

**Authors:** Naoki Watanabe, Tomohisa Watari, Yoshihito Otsuka, Tomoh Matsumiya

**Affiliations:** Hirosaki University Graduate School of Health Sciences, Hirosaki, Japan (N. Watanabe, T. Matsumiya); Kameda Medical Center, Kamogawa, Japan (T. Watari, Y. Otsuka)

**Keywords:** Klebsiella pneumoniae, bacteria, antimicrobial resistance, bacteremia, abscess, virulence factors, whole-genome sequencing, length of stay, drug resistance, bacterial infections, Japan

## Abstract

Hypervirulent *Klebsiella pneumoniae* (hvKp) is an emerging cause of invasive infection, but clinical relevance of a 5-biomarker genotypic definition remains unclear. We analyzed 207 episodes of *K. pneumoniae* bloodstream infection at a tertiary hospital in Japan during 2022–2024. We defined hvKp by PCR detection of all 5 biomarkers: *rmpA*, *rmpA2*, *iucA*, *iroB*, and *peg-344*. Overall, hvKp accounted for 14% (28/207) of episodes. Abscess complications occurred in 61% (17/28) of hvKp episodes and 13% (23/179) of classical *K. pneumoniae* episodes. hvKp case-patients had longer median hospitalization (28 vs. 14 days) and duration of antimicrobial drug treatment (43 vs. 14 days) than those with classical *K. pneumoniae*. Most (9/10) sequence type 23 hvKp episodes involved abscesses. Short-read whole-genome sequencing detected CTX-M–type extended-spectrum β-lactamase genes in 11% (3/28) of hvKp isolates, representing 1.4% (3/207) of all *K. pneumoniae* episodes. The 5-biomarker definition delineated a clinically distinct subgroup, demonstrating its value for genomic surveillance of emerging hvKp.

*Klebsiella pneumoniae* is a leading cause of bloodstream infection and was linked to ≈790,000 deaths globally in 2019 (*1*). Classical *K. pneumoniae* (cKp) predominantly causes infections in patients with underlying or immunocompromising conditions. Conversely, hypervirulent *K. pneumoniae* (hvKp) can cause invasive, metastatic infections, including liver abscess, endophthalmitis, and meningitis, even in persons without underlying conditions (*2*). Reports of hvKp acquiring carbapenem-resistance genes triggered alerts from the World Health Organization and European Centre for Disease Prevention and Control, highlighting the need for reliable identification of this pathotype (*3*,*4*).

Definitions of hvKp vary across studies, but the combination of 5 genotypic biomarkers (*iucA*, *iroB*, *peg-344*, *rmpA*, and *rmpA2*) has demonstrated high diagnostic accuracy for identifying hvKp (*5*–*7*). Among *K. pneumoniae* bloodstream infections, hvKp prevalence varies across geographic settings, from 4% in the United States and Norway to 26% in Japan (*8*–*10*). However, direct comparisons across studies are complicated by variations in hvKp definitions. Moreover, epidemiologic and clinical data specifically on the basis of the 5-biomarker definition remain limited. Consequently, whether 5 biomarker–defined hvKp identifies a clinically and epidemiologically distinct subgroup of bloodstream infections and whether its effects differ across lineages remain unclear.

We conducted a retrospective cohort study at a tertiary hospital in Japan to compare the clinical characteristics of 5 biomarker–defined hvKp and cKp bloodstream infections. The primary aim was to determine whether 5 biomarker–defined hvKp was more frequently associated with abscess complications. Secondary aims were to estimate post–disease onset length of stay (LOS; i.e., hospitalization), total antibiotic duration, and 30-day mortality risk. We also summarized the lineage-specific genomic features of hvKp isolates and assessed convergence between hypervirulence and antimicrobial resistance.

## Materials and Methods

### Study Design and Data Collection

This single-center, retrospective cohort study was conducted at Kameda Medical Center, a 907-bed tertiary hospital in Kamogawa, Japan. We included consecutive patients from whom *K. pneumoniae* species complex was isolated in >1 set of blood cultures during January 1, 2022–December 31, 2024. Patients with bloodstream infections had routine consultations for infectious diseases at the institution. For patients with multiple bloodstream infection episodes, we included only the first episode. 

Of 290 unique episodes of *K. pneumoniae* species complex bloodstream infections identified, we excluded 2 because clinical data were insufficient or no stored isolate was available (Figure 1). We excluded 81 other episodes because species-level differentiation by PCR (*11*) identified *K. variicola* or *K. quasipneumoniae* isolates. Thus, we had a total of 207 *K. pneumoniae* isolates for analysis. 

**Figure 1:**
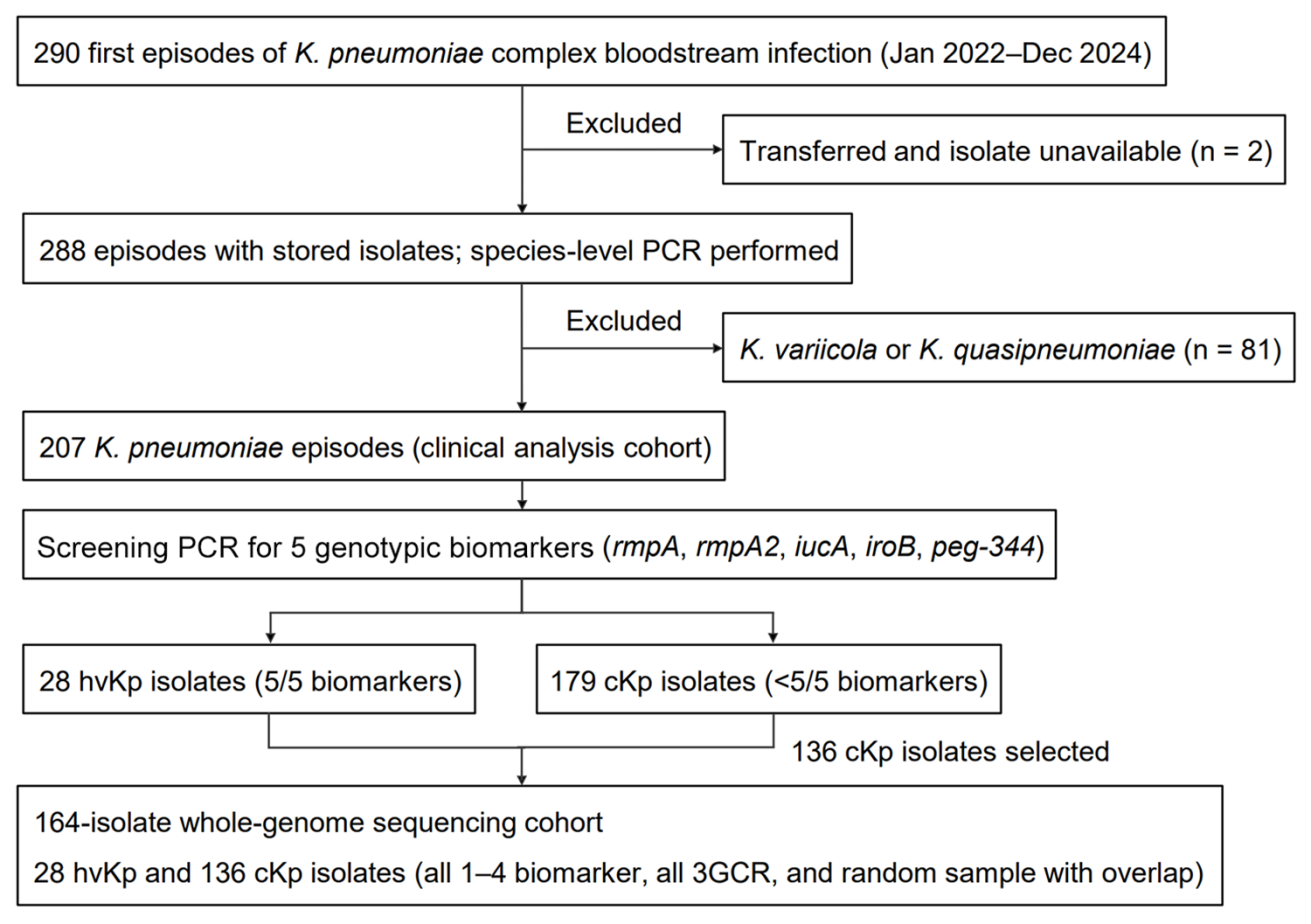
Flow diagram of episode inclusion in study of 5-biomarker definition for hvKp bloodstream infection and abscess complications, Japan, 2022–2024. Of 290 first episodes of *Klebsiella pneumoniae* complex bloodstream infection, 207 *K. pneumoniae* episodes were included in the clinical analysis. Whole-genome sequencing was performed on 164 isolates by using an enriched sampling strategy comprising all hvKp isolates, all isolates with 1–4 biomarkers, all 3GCR isolates, and a random sample of the cKp isolates with overlap between categories. 3GCR, third-generation cephalosporin–resistant; cKp, classical *K. pneumoniae*; hvKp, hypervirulent *K. pneumoniae*.

We collected clinical and microbiological data from electronic medical records and laboratory information systems and recorded patient sex according to medical records. We processed blood cultures by using the BD BACTEC FX system (Becton, Dickinson and Company, https://www.bd.com) and performed species identification by using a MALDI Biotyper (Bruker Daltonics GmbH, https://www.bruker.com) for matrix-assisted laser desorption/time-of-flight mass spectrometry. We used a Dry Plate Eiken (Eiken Chemical Co., Ltd., https://www.eiken.co.jp) to perform antimicrobial susceptibility testing by the broth microdilution method. We tested for susceptibility to the following antimicrobial agents: piperacillin, ampicillin/sulbactam, amoxicillin/clavulanate, piperacillin/tazobactam, cefazolin, ceftriaxone, cefepime, cefmetazole, imipenem, meropenem, aztreonam, gentamicin, tobramycin, amikacin, levofloxacin, ciprofloxacin, minocycline, and trimethoprim/sulfamethoxazole. We interpreted susceptibility results according to Clinical and Laboratory Standards Institute standards (*12*). This study was approved by the Research Ethics Committee of the Kameda Medical Center (approval no. 25-016) with a waiver for informed consent and an opt-out process.

### Definitions

We classified isolates as hvKp or cKp according to the 5-biomarker definition and assessed antimicrobial resistance phenotypes separately. We defined hvKp as presence of all 5 genotypic biomarkers (*iucA*, *iroB*, *peg-344*, *rmpA*, and *rmpA2*), as proposed by others (*5*–*7*). We classified isolates that did not meet the 5-biomarker definition as cKp. We performed PCR-based screening by using previously published primers for *rmpA*, *rmpA2*, *iucA*, *iroB*, and *peg-344* (*5*) (Appendix Table 1). We performed screening sequentially: first, we tested for *rmpA*; then, we tested for the other markers only in *rmpA*-positive isolates. We assigned the hvKp classification retrospectively for study analyses; the classification was not available to the treating clinicians during routine care. 

We defined third-generation cephalosporin resistance (3GCR) as nonsusceptibility to ceftriaxone and multidrug resistance as nonsusceptibility to >1 agent in >3 antimicrobial categories (*13*). We classified infection onset as community-acquired if the index blood culture was collected within 48 hours of admission without prior healthcare exposure. We classified all other episodes as healthcare-associated. We calculated Charlson comorbidity index (CCI) after excluding age points (*14*). We defined abscess complications only on the basis of imaging findings and excluded clinically suspected abscesses without imaging confirmation. We defined metastatic infection as a secondary infectious focus anatomically distinct from the presumed primary source of bloodstream infection, including abscesses and nonsuppurative manifestations, such as endophthalmitis. We defined post–disease onset LOS as the number of days from index blood culture collection to hospital discharge or transfer. We determined total antibiotic duration as the total number of days of antimicrobial therapy for a bloodstream infection episode and its complications, including oral therapy after discharge (Appendix). 

### Whole-Genome Sequencing

We performed whole-genome sequencing (WGS) on a subset of isolates selected to maximize representation of biomarker-positive and antimicrobial-resistant isolates within the available sequencing budget. The sequenced subset comprised 164 isolates, which included all hvKp isolates (n = 28), a random sample of cKp isolates (n = 125), additional cKp isolates selected to ensure complete representation of isolates with 1–4 positive biomarkers (n = 8), and 3GCR isolates (n = 31). Selection categories overlapped. 

We performed short-read sequencing on a NovaSeq X Plus platform (Illumina, https://www.illumina.com) and used Unicycler (*15*) to perform de novo assembly. We assessed assembly quality by using QUAST and CheckM2 with predefined quality criteria (*16*,*17*). 

We performed multilocus sequence typing, capsular locus typing, virulence gene profiling, and virulence scoring by using Kleborate version 3.2.4 outputs provided by Pathogenwatch (*18*,*19*). We additionally used AMRFinderPlus to evaluate β-lactam resistance determinants (*20*). We inferred a recombination-filtered core-genome single-nucleotide polymorphism (SNP) phylogeny by using Snippy (https://github.com/tseemann/snippy) to map reads to the *K. pneumoniae* NTUH-K2044 reference genome (GenBank accession no. AP006725.1), removed recombinant regions with Gubbins (*21*), and constructed a maximum-likelihood tree with IQ-TREE (*22*). We calculated pairwise SNP differences within selected lineages from the recombination-filtered core-genome SNP alignment. For hvKp isolates carrying extended-spectrum β-lactamase (ESBL) genes, we performed contig-level analysis to assess the genomic context of resistance and virulence determinants (Appendix). 

### Statistical Analysis

We used EZR version 1.70 (*23*) for statistical analyses. We compared continuous variables by using the Mann-Whitney U test and categorical variables by using Fisher exact tests. All tests were 2-sided, and we considered p<0.05 statistically significant.

We selected a priori covariates as baseline confounders on the basis of clinical relevance and constrained the number of covariates by the number of outcome events to avoid overfitting. We used multivariable logistic regression to evaluate abscess complications and adjusted for age, sex, and CCI. For 30-day mortality risk, we used Firth’s penalized logistic regression, adjusted for age and CCI because the number of death events was limited relative to the number of candidate covariates (*24*). 

For LOS and antibiotic duration, we fitted 2 sequential models: model A estimated the overall association of hvKp with each outcome, and model B included abscess complications to assess attenuation. We analyzed LOS as ln(LOS + 1) by using multivariable linear regression and exponentiated coefficients to report adjusted ratios with 95% CIs. We also analyzed total antibiotic duration among the treatment completers, using multivariable linear regression to analyze duration after log transformation. For both models, we used a common base adjustment set comprising age, sex, CCI, and infection acquisition category. For antibiotic duration, we also included the primary infection source because treatment duration was strongly influenced by the infectious syndrome and source-control requirements. For each outcome, we excluded episodes that had missing data from the corresponding analysis (Appendix). 

As a prespecified exploratory analysis, we stratified outcomes by biomarker counts of 0, 1–4, or all 5. We descriptively summarized genomic features of the hvKp isolates, including lineage, capsule type, and virulence plasmid structure, rather than formally comparing across lineages.

## Results

### Patient Characteristics

Among 207 *K. pneumoniae* bloodstream infection episodes, 28 (14% [95% CI 9%–19%]) were classified as hvKp and 179 (86% [95% CI 81%–91%]) as cKp (Table 1; Appendix Table 2). The median age was similar between the 2 groups: hvKp median patient age was 76 (interquartile range [IQR] 71–81) years, and cKp median patient age was 78 (IQR 69–85) years. In the hvKp group, 75% (21/28) of patients were male and 25% (7/28) were female, whereas the cKp group comprised 60% (108/179) male and 40% (71/179) female patients. Patients with hvKp had fewer underlying conditions; 57% (16/28) had CCI scores >2 compared with 80% (143/179) of the cKp group. Patients with hvKp also less frequently had markers of healthcare exposure than the cKp group, including indwelling devices (43% [12/28] vs. 64% [114/179]) and prior antimicrobial drug use (32% [9/28] vs. 55% [99/179]). 

**Table 1:** Baseline characteristics of 207 patients with hypervirulent and classical *Klebsiella pneumoniae* bloodstream infections in a study of five-biomarker definition for hvKp, Japan, 2022–2024*

Characteristics	hvKp, n = 28	cKp, n = 179	p value†
Demographic characteristics			
Median age, y (IQR)	76 (71–81)	78 (69–85)	0.64
Sex			
M	21 (75)	108 (60)	0.15
F	7 (25)	71 (40)	
Median body mass index (IQR), n = 201	21 (20–23)	20 (18–24)	0.65
Underlying conditions			
Median CCI score (IQR)	2 (1–4)	3 (2–5)	0.15
Diabetes, any	9 (32)	62 (35)	>0.99
Chronic kidney disease	3 (11)	23 (13)	>0.99
Liver disease	1 (4)	9 (5)	>0.99
Solid tumor	8 (29)	64 (36)	0.53
Healthcare exposure			
Indwelling device	12 (43)	114 (64)	0.04
Recent hospitalization	12 (43)	112 (63)	0.06
Prior antibiotic exposure	9 (32)	99 (55)	0.02
Infection characteristics			
Onset classification			0.30
Healthcare-associated	15 (54)	114 (64)	
Community-acquired	13 (46)	65 (36)	
Primary source of infection			0.03
Urinary tract	7 (25)	37 (21)	
Catheter-related	0 (0.0)	5 (3)	
Intraabdominal	13 (46)	83 (46)	
Respiratory	4 (14)	5 (3)	
Other	1 (4)	1 (<1)	
Unknown	3 (11)	48 (27)	
Microbiological characteristics			
Multidrug resistance	3 (11)	53 (30)	0.04
Polymicrobial bloodstream infection	3 (11)	42 (23)	0.15

*Values are no. (%) except as indicated. CCI, Charlson comorbidity index; cKp, classical *K. pneumoniae*; hvKp, hypervirulent *K. pneumoniae*; IQR, interquartile range. †p values were calculated using Fisher exact test for categorical variables and Mann–Whitney U test for continuous variables and were provided for descriptive purposes.

Primary infection sources were broadly similar between the groups, although respiratory infection was more frequent in the hvKp group and unknown source was more frequently reported for the cKp group. However, respiratory infection remained uncommon, accounting for only 4% (9/207) of episodes. Polymicrobial bloodstream infection was less frequent among hvKp episodes than among cKp episodes (11% [3/28] vs. 23% [42/179]). Multidrug resistance was less common in the hvKp group than in the cKp group (11% [3/28] vs. 30% [53/179]) (Appendix Table 3). Appropriate empiric antimicrobial therapy was administered in 27 (96%) of the hvKp episodes and 151 (84%) of the cKp episodes. We compiled data and population analyses (Appendix Table 4).

### Abscess Complications

Abscess complications occurred in 40 (19%) *K. pneumoniae* episodes, among which 23 (58%) were cKp and 17 (42%) were hvKp according to the 5-biomarker definition. The within-group frequency of abscess complications was 61% (95% CI 41%–79%) in hvKp episodes and 13% (95% CI 8%–19%) in cKp episodes (Table 2). Among episodes with abscess complications, polymicrobial bloodstream infection was observed in 12% (2/17) of hvKp episodes and 26% (6/23) of cKp episodes. In the multivariable logistic regression analysis, hvKp was independently associated with abscess complications (adjusted odds ratio [aOR] 10.7 [95% CI 4.4–26.2]) (Appendix Table 5). 

**Table 2:** Clinical outcomes and adjusted associations among *Klebsiella pneumoniae* bloodstream infection patients in a study of 5-biomarker definition for hvKp bloodstream infection and abscess complications, Japan, 2022–2024*

Clinical outcome	hvKp, n = 28	cKp, n = 179	Total no.	Estimate (95% CI)†	
				Crude	Adjusted
Any abscess complication	17 (61)	23 (13)	207 (100)	10.5 (4.4–25.2)	10.7 (4.4–26.2)
Liver abscess	9 (32)	12 (7)	207 (100)	–	–
Other abscess sites‡	11 (39)	12 (7)	207 (100)	–	–
Metastatic infection	4 (14)	1 (<1)	207 (100)	–	–
Median postonset hospitalization, d (IQR)§#	28 (14–42)	14 (11–18)	206 (99.5)	1.6 (1.2–2.2)	Model A, 1.6 (1.2–2.2)
					Model B, 1.1 (0.8–1.5)
Median antibiotic duration, d (IQR)#††	43 (16–67)	14 (14–18)	168 (81)	2.1 (1.6–2.7)	Model A, 2.1 (1.6–2.8)
					Model B, 1.2 (1.0–1.5)
30-d mortality rate, deaths/no.	4/27 (15)	34/170 (20)	197 (95)	0.8 (0.2–2.1)	0.9 (0.3–2.4)

*Data are no. (%) or median (IQR) unless otherwise indicated. Adjusted estimate is shown only for prespecified primary, secondary, and supportive outcomes. Liver abscess, other abscess sites, and metastatic infection are presented descriptively and were not modeled separately. cKp, classical *K. pneumoniae*; hvKp, hypervirulent *K. pneumoniae*; IQR, interquartile range. †Estimates are odds ratios for abscess complications and 30-day mortality, and ratios for length of hospitalization and antibiotic duration. Adjusted estimates for abscess complication were derived from multivariable logistic regression adjusted for age, sex, and Charlson comorbidity index (CCI); for post–disease onset length of stay, from multivariable linear regression of ln(length of stay + 1) adjusted for age, sex, CCI, and infection acquisition category; for total antibiotic duration, from multivariable linear regression of log-transformed duration among treatment completers adjusted for age, sex, CCI, infection acquisition category, and primary infection source; and for 30-d mortality, from Firth penalized logistic regression adjusted for age and CCI. ‡Other abscess sites included pelvic or intra-abdominal abscess, lung abscess or necrotizing pneumonia, prostatic abscess, endophthalmitis, central nervous system abscess, and musculoskeletal or soft tissue infection; episodes may have had >1 site. §Post–disease onset length of stay was defined as days from index blood culture collection to hospital discharge or interhospital transfer. If hospitalization continued for reasons judged unrelated to the bloodstream infection, follow-up was censored at the end of the bloodstream infection-directed therapy. Episodes ending in in-hospital death were included, with length of stay calculated up to the date of death. #Model A indicates the base-adjusted model and model B indicates the model additionally adjusted for the development of an abscess complication. ††Total antibiotic duration was analyzed among treatment completers.

The most common hvKp abscess site was the liver (32% [9/28], compared with 7% [12/179] in cKp infections), and hvKp also more frequently involved abscesses at other sites than cKp infections (39% [11/28] vs. 7% [12/179]). Extrahepatic sites included urogenital, intraabdominal, central nervous system, pulmonary, musculoskeletal, and ocular locations, some of which exclusively occurred in hvKp (Appendix Table 2). We identified metastatic infection in 14% (4/28) of hvKp and <1% (1/179) of cKp episodes. Abscess complication rates rose with the number of biomarkers, occurring in 12% (21/171) of the 0-marker group, 25% (2/8) of the 1–4-marker group, and 61% (17/28) of the 5-marker group (Figure 2; Appendix Table 6).

**Figure 2:**
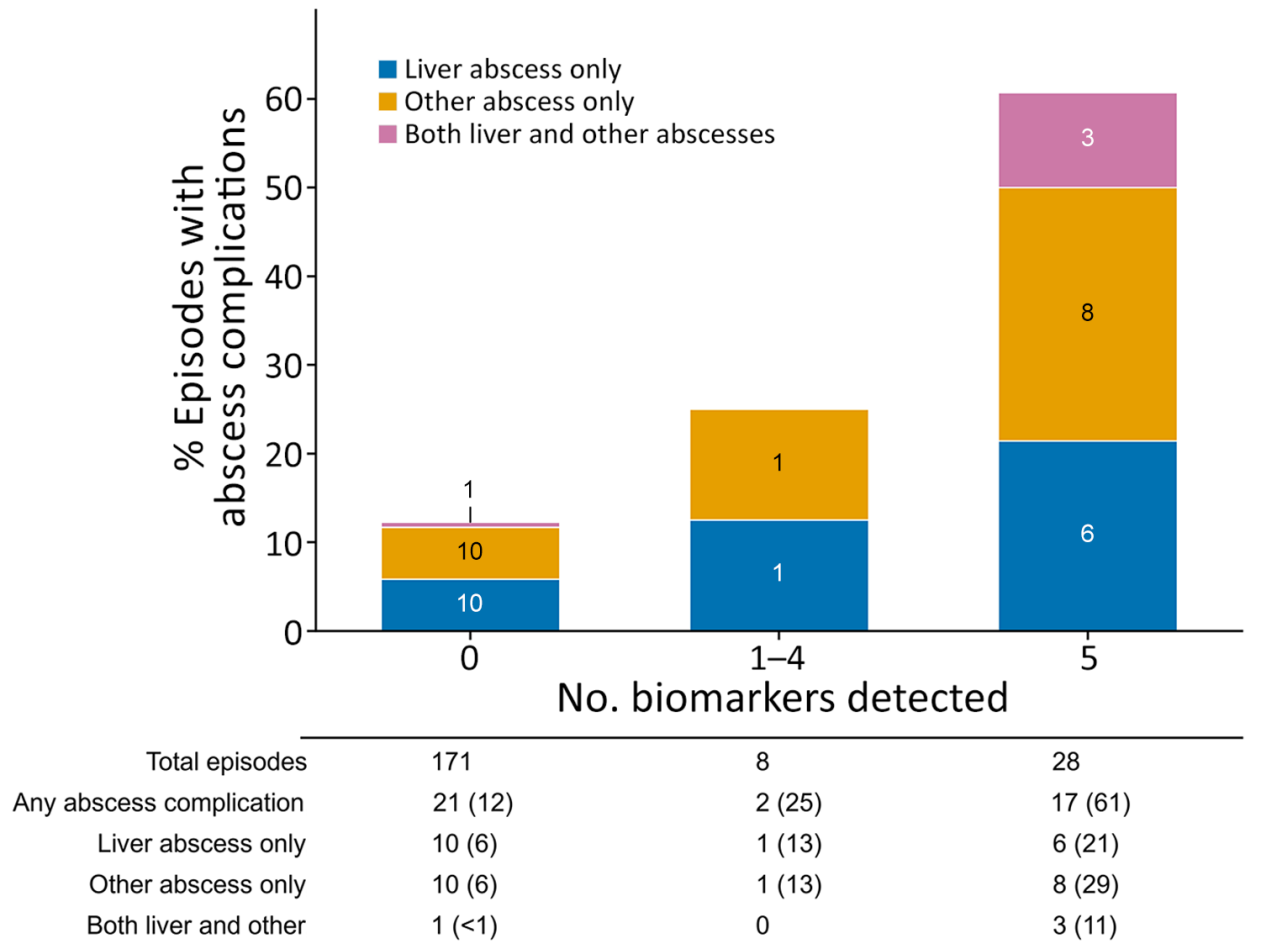
Abscess complications among 207 episodes of *Klebsiella pneumoniae* bloodstream infection by biomarker group in study of hypervirulent *K. pneumoniae*, Japan, 2022–2024. Stacked bars show mutually exclusive categories of liver abscess only, nonhepatic abscess only, and both liver and nonhepatic abscesses. Bar height represents the percentage of episodes with any abscess complication; numbers within colored bar segments indicate case counts. Case counts and percentages are provided below each bar.

### LOS, Antibiotic Duration, and 30-Day Mortality Risk

Among 207 episodes, 206 (28 hvKp and 178 cKp) had post–disease onset LOS data available. LOS was longer for the hvKp group (median 28 [IQR 14–42] days) than for the cKp group (14 [IQR 11–18] days). For antibiotic duration, we excluded 39 episodes, primarily because of patient death before treatment completion. Among the 168 (24 hvKp and 144 cKp) patients who completed treatment, the total antibiotic duration was longer in the hvKp group (median 43 [IQR 16–67] days) than in the cKp group (median 14 [IQR 14–18] days). In the adjusted analyses, hvKp was associated with longer LOS (adjusted ratio 1.6 [95% CI 1.2–2.2]) and antibiotic duration (adjusted ratio 2.1 [95% CI 1.6–2.8]) (Appendix Tables 7, 8). Those associations were attenuated after additional adjustment for abscess complication (LOS adjusted ratio 1.1 [95% CI 0.8–1.5]; antibiotic duration adjusted ratio 1.2 [95% CI 1.0–1.5]). The prespecified threshold analyses of prolonged antibiotic duration aligned with those findings (Appendix Table 9).

The 30-day mortality rate was 15% (95% CI 4%–34%) in the hvKp group and 20% (95% CI 14%–27%) in the cKp group; we excluded episodes with unknown vital status. In the adjusted analyses, the aOR was 0.9 (95% CI 0.3–2.4) for 30-day mortality associated with hvKp (Appendix Table 10). Intensive care unit admission rates were 18% (5/28) in the hvKp group and 14% (25/179) in the cKp group. Vasopressor use was 25% (7/28) in the hvKp group and 28% (51/179) in the cKp group. Mechanical ventilation was more frequent in the hvKp group (14% [4/28]) compared with the cKp group (3% [5/179]); nonetheless, we did not perform an adjusted analysis on those categories because so few events were recorded.

### Genomic Characteristics and Lineage-Specific Findings

We performed WGS on 164 isolates, including 28 hvKp and 136 cKp isolates. All assemblies met predefined quality criteria. Patient clinical characteristics were similar between those with randomly sampled cKp isolates (n = 125) and those cKp isolates not included in the random sample (n = 54), except for the distribution of primary infection sources (Appendix Tables 11, 12). Kleborate virulence scores, based on key virulence loci (*19*), were >3 in all 28 sequenced hvKp isolates: 7 had a score of 3; 6 had a score of 4; and 15 had a score of 5. Among sequenced cKp isolates, 98% (133/136) had scores of 0–2, whereas 3 had a score of 4 despite not meeting the 5-biomarker definition.

We analyzed sequence type (ST) and capsule synthesis locus (K-locus [KL]) distributions among isolates (Appendix Tables 13, 14). Among hvKp isolates, predominant lineages were ST23/KL1 (36%, 10/28) and ST65/KL2 (29%, 8/28) (Figure 3), within which pairwise SNP differences ranged from 36 to 359 among the 10 ST23/KL1 isolates and from 132 to 432 among the 8 ST65/KL2 isolates (Appendix Figure). Other hvKp lineages included ST412/KL57 accounting for 14% (4/28) and ST86/KL2 accounting for 7% (2/28) of hvKp isolates. Randomly sampled cKp isolates comprised 75 sequence types, most commonly ST37 (8%, 10/125) and ST45 (7%, 9/125). Among the 3GCR isolates, 3 lineages predominated: ST307/KL102 (26%, 8/31), ST37/KL15 (23%, 7/31), and ST353/KL110 (23%, 7/31). 

**Figure 3:**
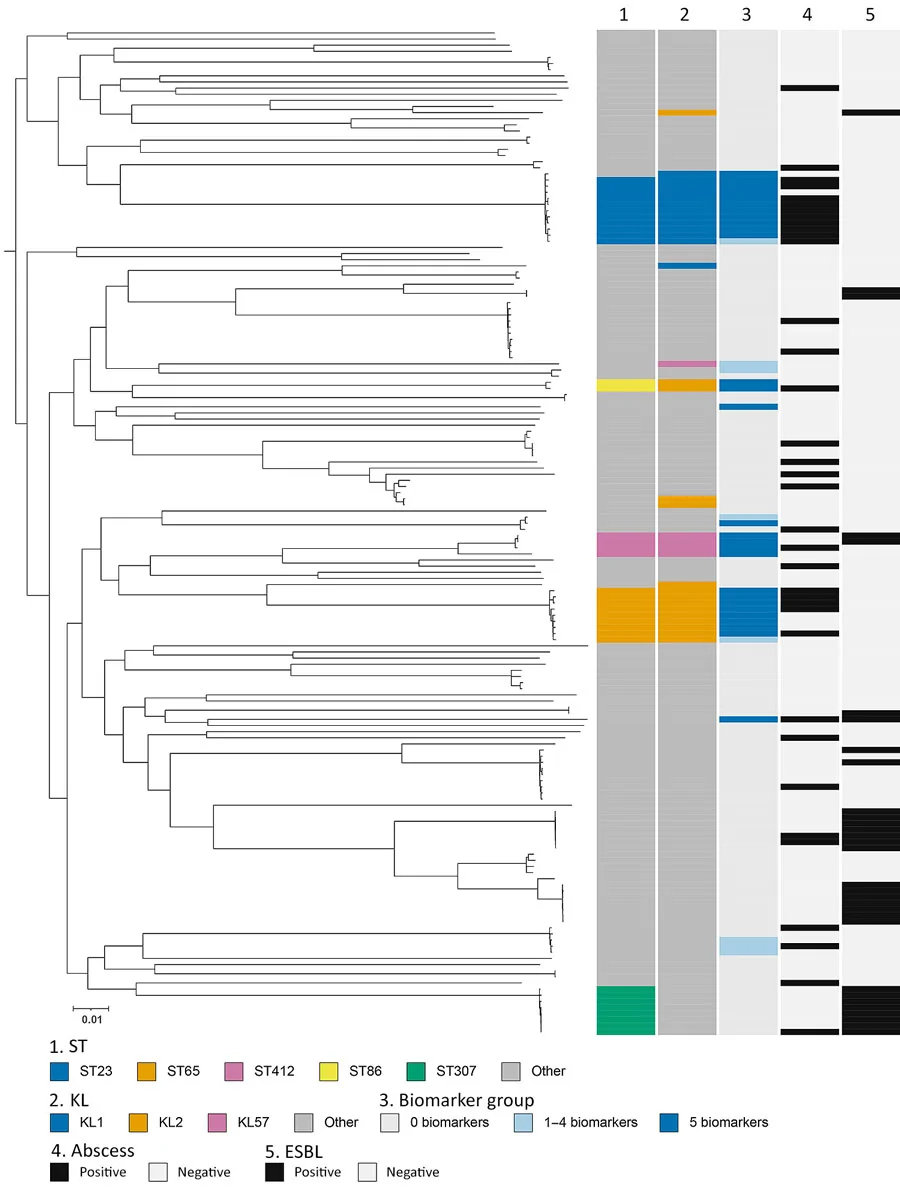
Maximum-likelihood phylogeny of 164 isolates from study of 5-biomarker definition for hypervirulent *Klebsiella pneumoniae* bloodstream infection and abscess complications, Japan, 2022–2024. The tree was inferred from a recombination-filtered core genome single-nucleotide polymorphism alignment. Annotation tracks indicate the ST, KL capsule type, biomarker group, abscess complication, and ESBL status. ESBL, extended-spectrum β-lactamase; KL, K-locus; ST, sequence type. Scale bar indicates nucleotide substitutions per site.

STs commonly associated with hvKp were uncommon among 136 sequenced cKp isolates; ST23/KL1 and ST65/KL2 were each detected in 1 isolate, and ST86 was not detected. Both ST23/KL1 and ST65/KL2 cKp isolates were in the 1–4 biomarker group and carried 3 of the 5 biomarkers, *rmpA*, *iroB*, and *peg-344*.

Abscess complication rates among hvKp episodes differed by lineage (Table 3): 9/10 (90%) in ST23/KL1, 5/8 (63%) in ST65/KL2, and 1/4 (25%) in ST412/KL57. We also observed genomic differences across hvKp lineages. ST23/KL1 isolates uniformly carried intact *K. pneumoniae* virulence plasmid 1 (KpVP-1) and yersiniabactin, whereas most (88%, 7/8) ST65/KL2 isolates had KpVP-1 truncation.

**Table 3:** Lineage-specific genomic and clinical features of hypervirulent *Klebsiella pneumoniae* isolates from cases of bloodstream infection and abscess complications, Japan, 2022–2024*

Lineage	No. isolates	K-locus	KpVP-1 intact	ybt-positive	ESBL-positive	HAI	Abscess
ST23	10	KL1	10 (100)	10 (100)	0	2 (20)	9 (90)
ST65	8	KL2	1 (13)	3 (38)	0	5 (63)	5 (63)
ST412	4	KL57	2 (50)	2 (50)	2 (50)	3 (75)	1 (25)
ST86	2	KL2	2 (100)	2 (100)	0	1 (50)	1 (50)
Other†	4	KL1, KL20, KL54	4 (100)	4 (100)	1 (25)	4 (100)	1 (25)

*Values are no. (%) except as indicated. ESBL, extended-spectrum β-lactamase; HAI, healthcare-associated infection; KpVP-1, *K. pneumoniae* virulence plasmid 1; ST, sequence type; ybt, yersiniabactin locus. †Other lineages included ST268 (n = 1), ST29 (n = 1), ST893 (n = 1), and a single-locus variant of ST23 (n = 1).

We identified ESBL genes in 11% (3/28) of hvKp episodes, representing 1.4% (3/207) of all *K. pneumoniae* bloodstream infection episodes. Two episodes involved ST412/KL57 isolates harboring *bla*_CTX-M-15_; those isolates differed by 23 recombination-filtered core-genome SNPs and were isolated in 2023 and 2024. The third episode involved an ST893/KL20 isolate carrying *bla*_CTX-M-55_. All 3 convergence episodes were healthcare-associated, and none resulted in death. In all 3 isolates, the *bla*_CTX-M_ gene was located on a contig assigned to a putative plasmid cluster, separate from the contigs carrying virulence-associated sequences (Appendix). We did not detect any carbapenemase genes among the 164 sequenced isolates.

## Discussion

In this retrospective cohort of 207 *K. pneumoniae* bloodstream infection episodes, we identified 28 (14%) hvKp episodes by using the 5-biomarker definition. Those episodes were characterized by frequent abscess complications and prolonged hospitalization and antibiotic treatment. WGS revealed genomic heterogeneity within hvKp and lineage-specific differences in abscess frequency.

The 14% prevalence of hvKp in this cohort was lower than that reported from Japan (*10*) but was higher than that reported in the United States and Norway (*9*,*25*). Those differences likely reflect both the epidemiologic setting and variability in hvKp definitions across studies. Broader definitions on the basis of a subset of biomarkers might classify isolates in the 1–4-marker group as hvKp, thereby yielding higher prevalence estimates than the 5-marker definition used in this study. In this cohort, abscess complication rates rose across biomarker strata, from 12% in the 0-marker group to 25% in the 1–4-marker group and 61% in the 5-marker group. That gradient aligns with studies reporting that requiring all 5 biomarkers provides greater accuracy for identifying hvKp than lower thresholds (*6*). Therefore, isolates positive for 1–4 markers might be better considered an intermediate group rather than definitively considered hvKp.

 The 5-biomarker definition identified an abscess-prone subgroup of *K. pneumoniae* bloodstream infections. However, 58% of abscess complications occurred in cKp episodes. That finding underscores that the 5-biomarker definition identifies a subgroup at increased risk for abscess complications rather than a criterion intended to capture all abscess complications. Although the observed 30-day mortality risk was not higher in hvKp than cKp episodes, that difference should not be interpreted as evidence that hvKp imposes no clinically meaningful burden. Rather, the burden was reflected in longer hospitalization and antibiotic treatment. The discrepancy with prior reports of increased early hvKp mortality (*26*) might be explained by differences in infection–source composition. In our cohort, respiratory infections, previously associated with poor outcomes in *K. pneumoniae* bloodstream infection (*8*), accounted for only 4% of episodes. Mortality rates for hvKp might depend on clinical setting and source distribution, whereas abscess-associated treatment burden might occur even in the absence of higher mortality rates.

The clinical and public health challenges posed by *K. pneumoniae* bloodstream infections vary with the local prevalence of hvKp. In a cohort from the United States, where hvKp is rare, resistance to the initial prescribed antibiotic was an independent predictor of in-hospital death (*8*). In our analysis, antimicrobial resistance remained a concern for cKp, which accounted for 86% of episodes and had a higher multidrug-resistance rate than hvKp. Conversely, hvKp isolates were generally susceptible to standard empiric agents but were associated with abscess complications and prolonged care. Those findings suggest that management priorities could differ between subgroups: cKp warrants attention to antimicrobial resistance, whereas hvKp might warrant careful evaluation for abscess complications and source control. Thus, awareness of local epidemiology could help align management strategies with the dominant clinical burden of *K. pneumoniae* bloodstream infection.

Beyond the clinical burden of hvKp bloodstream infection, clinically accessible diagnostic tests and ongoing genomic surveillance remain priorities. Currently, the 5-biomarker PCR for hvKp is a research tool, and no commercial assay is available for routine clinical use (*5*,*7*). Although such assays could be developed on existing PCR platforms (*27*), they have not yet been validated in clinical laboratories (*7*). Distinguishing hvKp from cKp could enable abscess evaluation and source control. Another concern is the genomic convergence of hypervirulence and antimicrobial resistance (*3*). A US multicenter study found that 0.7% of carbapenem-resistant *K. pneumoniae* isolates harbored a virulence plasmid (*28*). Although we did not identify carbapenemase-producing isolates in our cohort, 11% (3/28) of hvKp episodes were ESBL-producing, corresponding to 1.4% (3/207) of all *K. pneumoniae* bloodstream infection episodes studied. In all 3 ESBL-producing hvKp isolates, *bla*_CTX-M_ genes were carried on plasmid contigs separate from the virulence-associated sequences, suggesting independent acquisition. Collectively, those findings support the need for clinically accessible hvKp diagnostic tools and continued genomic surveillance to monitor the convergence of hypervirulence and antimicrobial resistance.

The first limitation of this study is that the retrospective design at a single tertiary hospital might limit the generalizability of our findings. Second, abscess imaging was performed at the discretion of the treating clinicians without a standardized study protocol, and imaging-frequency data were unavailable. Because hvKp episodes might more frequently have features prompting cross-sectional imaging, differential ascertainment of abscess complications cannot be excluded and could have contributed to the observed intergroup differences in abscess rates. Nevertheless, the large effect size and descriptive gradient in abscess frequency across biomarker strata suggest that ascertainment bias alone is unlikely to fully account for the observed association. Third, the number of hvKp episodes was small, limiting the statistical power of some comparisons, including 30-day mortality rate. Fourth, WGS was performed on 164 of 207 isolates. The clinical characteristics were similar between randomly sampled cKp isolates and those not included in the random sample; however, residual selection bias cannot be ruled out. Despite those limitations, the consecutive case design in a tertiary care setting with routine infectious diseases consultation might have enabled characterization of the clinical burden of abscesses in this cohort.

In conclusion, we identified 5-biomarker–defined hvKp as a clinically and epidemiologically distinct subgroup of *K. pneumoniae* bloodstream infection characterized by frequent abscess complications and prolonged care in a tertiary-care cohort in Japan. The clinical burden differed between groups, with abscess-prone disease in hvKp and greater antimicrobial resistance in cKp. Those findings suggest that clinical management should be informed by local epidemiology and support the need for continued surveillance of hvKp and cKp in bloodstream infections.

Appendix Additional information on five-biomarker definition for hypervirulent *Klebsiella pneumoniae* bloodstream infection and abscess complications, Japan, 2022–2024.

## References

[1] GBD 2019 Antimicrobial Resistance Collaborators. Global mortality associated with 33 bacterial pathogens in 2019: a systematic analysis for the Global Burden of Disease Study 2019. Lancet. 2022;400:2221–48. 10.1016/S0140-6736(22)02185-7 36423648 PMC9763654

[2] Russo TA, Marr CM. Hypervirulent *Klebsiella pneumoniae.* Clin Microbiol Rev. 2019;32:e00001–00019. 10.1128/CMR.00001-19 31092506 PMC6589860

[3] World Health Organization. Antimicrobial resistance, hypervirulent *Klebsiella pneumoniae*—global situation. 2024 Jul 31 [cited 2024 Aug 3]. https://www.who.int/emergencies/disease-outbreak-news/item/2024-DON527

[4] European Centre for Disease Prevention and Control. Rapid risk assessment—emergence of hypervirulent *Klebsiella pneumoniae* ST23 carrying carbapenemase genes in EU/EEA countries—first update. 2024 Feb 14 [cited 2026 Mar 13]. https://www.ecdc.europa.eu/en/publications-data/risk-assessment-emergence-hypervirulent-Klebsiella-pneumoniae-eu-eea

[5] Russo TA, Olson R, Fang CT, Stoesser N, Miller M, MacDonald U, et al. Identification of biomarkers for differentiation of hypervirulent *Klebsiella pneumoniae* from classical *K. pneumoniae.* J Clin Microbiol. 2018;56:e00776–18. 10.1128/JCM.00776-18 29925642 PMC6113484

[6] Russo TA, Alvarado CL, Davies CJ, Drayer ZJ, Carlino-MacDonald U, Hutson A, et al. Differentiation of hypervirulent and classical *Klebsiella pneumoniae* with acquired drug resistance. MBio. 2024;15:e0286723. 10.1128/mbio.02867-23 38231533 PMC10865842

[7] Russo TA, Lebreton F, McGann PT. A step forward in hypervirulent *Klebsiella pneumoniae* diagnostics. Emerg Infect Dis. 2025;31:1–3. 10.3201/eid3101.241516 39714290 PMC11682795

[8] Roach DJ, Sridhar S, Oliver E, Rao SR, Slater DM, Hwang W, et al. Clinical and genomic characterization of a cohort of patients with *Klebsiella pneumoniae* bloodstream infection. Clin Infect Dis. 2024;78:31–9. 10.1093/cid/ciad507 37633257 PMC10810715

[9] Fostervold A, Raffelsberger N, Hetland MAK, Bakksjø R, Bernhoff E, Samuelsen Ø, et al. Assoc. on behalf of The Norwegian Study Group on *Klebsiella pneumoniae*. Risk of death in *Klebsiella pneumoniae* bloodstream infections is associated with specific phylogenetic lineages. J Infect. 2024;88:106155. 10.1016/j.jinf.2024.106155 38574775

[10] Harada S, Aoki K, Yamamoto S, Ishii Y, Sekiya N, Kurai H, et al. Clinical and molecular characteristics of *Klebsiella pneumoniae* isolates causing bloodstream infections in Japan: occurrence of hypervirulent infections in health care. J Clin Microbiol. 2019;57:e01206–19. 10.1128/JCM.01206-19 31434721 PMC6812994

[11] Barrios-Camacho H, Silva-Sánchez J, Cercas-Ayala E, Lozano-Aguirre L, Duran-Bedolla J, Aguilar-Vera A, et al. PCR system for the correct differentiation of the main bacterial species of the *Klebsiella pneumoniae* complex. Arch Microbiol. 2022;204:73. 10.1007/s00203-021-02668-x 34951665

[12] Clinical and Laboratory Standards Institute. Performance standards for antimicrobial susceptibility testing, 34th edition (M100-S34). Wayne (PA): The Institute; 2024.

[13] Magiorakos AP, Srinivasan A, Carey RB, Carmeli Y, Falagas ME, Giske CG, et al. Multidrug-resistant, extensively drug-resistant and pandrug-resistant bacteria: an international expert proposal for interim standard definitions for acquired resistance. Clin Microbiol Infect. 2012;18:268–81. 10.1111/j.1469-0691.2011.03570.x 21793988

[14] Charlson ME, Pompei P, Ales KL, MacKenzie CR. A new method of classifying prognostic comorbidity in longitudinal studies: development and validation. J Chronic Dis. 1987;40:373–83. 10.1016/0021-9681(87)90171-8 3558716

[15] Wick RR, Judd LM, Gorrie CL, Holt KE. Unicycler: Resolving bacterial genome assemblies from short and long sequencing reads. PLOS Comput Biol. 2017;13:e1005595. 10.1371/journal.pcbi.1005595 28594827 PMC5481147

[16] Gurevich A, Saveliev V, Vyahhi N, Tesler G. QUAST: quality assessment tool for genome assemblies. Bioinformatics. 2013;29:1072–5. 10.1093/bioinformatics/btt086 23422339 PMC3624806

[17] Chklovski A, Parks DH, Woodcroft BJ, Tyson GW. CheckM2: a rapid, scalable and accurate tool for assessing microbial genome quality using machine learning. Nat Methods. 2023;20:1203–12. 10.1038/s41592-023-01940-w 37500759

[18] Argimón S, David S, Underwood A, Abrudan M, Wheeler NE, Kekre M, et al.; NIHR Global Health Research Unit on Genomic Surveillance of Antimicrobial Resistance. Rapid genomic characterization and global surveillance of *Klebsiella* using Pathogenwatch. Clin Infect Dis. 2021;73:S325–35. 10.1093/cid/ciab784 34850838 PMC8634497

[19] Lam MMC, Wick RR, Watts SC, Cerdeira LT, Wyres KL, Holt KE. A genomic surveillance framework and genotyping tool for *Klebsiella pneumoniae* and its related species complex. Nat Commun. 2021;12:4188. 10.1038/s41467-021-24448-3 34234121 PMC8263825

[20] Feldgarden M, Brover V, Gonzalez-Escalona N, Frye JG, Haendiges J, Haft DH, et al. AMRFinderPlus and the Reference Gene Catalog facilitate examination of the genomic links among antimicrobial resistance, stress response, and virulence. Sci Rep. 2021;11:12728. 10.1038/s41598-021-91456-0 34135355 PMC8208984

[21] Croucher NJ, Page AJ, Connor TR, Delaney AJ, Keane JA, Bentley SD, et al. Rapid phylogenetic analysis of large samples of recombinant bacterial whole genome sequences using Gubbins. Nucleic Acids Res. 2015;43:e15. 10.1093/nar/gku1196 25414349 PMC4330336

[22] Wong TKF, Ly-Trong N, Ren H, Demotte P, Baños H, Roger AJ, et al. IQ-TREE 3: phylogenomic inference software using complex evolutionary models. Mol Biol Evol. 2026;43:msag117. 10.1093/molbev/msag117 42085559 PMC13191116

[23] Kanda Y. Investigation of the freely available easy-to-use software ‘EZR’ for medical statistics. Bone Marrow Transplant. 2013;48:452–8. 10.1038/bmt.2012.244 23208313 PMC3590441

[24] Firth D. Bias reduction of maximum likelihood estimates. Biometrika. 1993;80:27–38. 10.1093/biomet/80.1.27

[25] Kochan TJ, Nozick SH, Medernach RL, Cheung BH, Gatesy SWM, Lebrun-Corbin M, et al. Genomic surveillance for multidrug-resistant or hypervirulent *Klebsiella pneumoniae* among United States bloodstream isolates. BMC Infect Dis. 2022;22:603. 10.1186/s12879-022-07558-1 35799130 PMC9263067

[26] Tang Y, Du P, Du C, Yang P, Shen N, Russo TA, et al. Genomically defined hypervirulent *Klebsiella pneumoniae* contributed to early-onset increased mortality. Nat Commun. 2025;16:2096. 10.1038/s41467-025-57379-4 40025046 PMC11873152

[27] Yu F, Lv J, Niu S, Du H, Tang YW, Pitout JDD, et al. Multiplex PCR analysis for rapid detection of *Klebsiella pneumoniae* carbapenem-resistant (sequence type 258 [ST258] and ST11) and hypervirulent (ST23, ST65, ST86, and ST375) strains. J Clin Microbiol. 2018;56:e00731–18. 10.1128/JCM.00731-18 29925644 PMC6113471

[28] Jiang J, Long T, Porter AR, Lovey A, Lee A, Jacob JT, et al. Carbapenem-resistant, virulence plasmid–harboring *Klebsiella pneumoniae*, United States. Emerg Infect Dis. 2025;31:761–71. 10.3201/eid3104.241396 40072602 PMC11950267

