# Supplementary material for: Five-Biomarker Definition for Hypervirulent Klebsiella pneumoniae Bloodstream Infection and Abscess Complications, Japan, 2022–2024
Source: Emerg Infect Dis. 2026 Nov;32(11):1855–64. doi: 10.3201/eid3211.260825 (PMC13715868; doi:10.3201/eid3211.260825)
Supplement: Supplementary file 1 [file 26-0825-Techapp-s1.pdf]

Article DOI: https://doi.org/10.3201/eid3211.260825

EID cannot ensure accessibility for supplementary materials supplied by authors. Readers who have difficulty accessing supplementary content should contact the authors for assistance.

Five-Biomarker Definition for Hypervirulent *Klebsiella pneumoniae* Bloodstream Infection and Abscess Complications, Japan, 2022–2024

Appendix

Supplementary Methods Microbiological methods

Blood cultures were processed using the BD BACTEC FX system (BD, https://www.bd.com). Species identification was performed using MALDI Biotyper with the MBT Compass Library version 13 (Bruker Daltonics GmbH, https://www.bruker.com). Species-level differentiation within the *Klebsiella pneumoniae* species complex was performed by PCR as previously described (*1*).

PCR detection of 5 biomarkers

PCR-based screening was performed for the 5 biomarkers: *rmpA*, *rmpA2*, *iucA*, *iroB*, and *peg-344*. Screening was performed sequentially. All isolates were first tested for *rmpA*, and *rmpA*-positive isolates were then tested for *rmpA2*, *iucA*, *iroB*, and *peg-344*.

PCR assays were performed in 50-µL reaction mixtures containing 1× Ex Taq Buffer, 0.2 mM each dNTP, 0.4 µM of each primer, ≈1.25 U of TaKaRa Ex Taq® Hot Start Version (Takara Bio, https://www.takara-bio.com), and genomic DNA. Cycling conditions consisted of initial denaturation at 98°C for 10 s, 25 amplification cycles, and final extension at 72°C for 10 min. Primer sequences, target-specific annealing temperatures, extension times, expected product sizes, and source references are shown in the primer table below. These primer sequences and target-specific PCR conditions were based on Russo et al. (*2*), Supplementary Appendix Table 1.

PCR products were analyzed by electrophoresis on 2% TAE agarose gels and visualized by ethidium bromide staining. Bands corresponding to the expected product sizes were considered positive.

Clinical definitions

Infection onset was classified as community-acquired if the index blood culture was collected before or within 48 hours of hospital admission in the absence of prior healthcare exposure. All other episodes were classified as healthcare-associated, including those with onset

more than 48 hours after admission and those with onset within 48 hours in the presence of prior healthcare exposure. Healthcare exposure was defined as one or more of the following: outpatient chemotherapy, hemodialysis, outpatient intravenous therapy or wound care, residence in a long-term care facility, or presence of an indwelling vascular access device.

Appropriate empiric therapy was defined as initial empiric antimicrobial therapy that tested susceptible or susceptible-dose dependent against the causative isolate on antimicrobial susceptibility testing. Post-onset length of stay was defined as days from the index blood culture collection to hospital discharge or interhospital transfer. If hospitalization continued for reasons judged unrelated to the bloodstream infection, follow-up was censored at the end of bloodstream infection-directed therapy. Episodes ending in in-hospital death were included, with length of stay calculated to death. Total antibiotic duration was defined as the total number of days of antimicrobial therapy administered for the bloodstream infection episode and its complications, including oral therapy prescribed after hospital discharge.

Missing data and analysis populations

The primary outcome, abscess complication, was assessed in all included episodes. Thirty-day mortality was analyzed among episodes with available 30-day vital status. Post-onset length of stay was analyzed among episodes with available follow-up data. Total antibiotic duration was analyzed among treatment completers with available duration data; episodes ending

in death before completion of therapy were excluded from the primary antibiotic-duration analysis.

Whole-genome sequencing

Genomic DNA was extracted using the MagLEAD instrument with the manufacturer's kit (Precision System Science, https://www.pss.co.jp). Short-read sequencing libraries were prepared using the Illumina DNA Prep (M) Tagmentation (Illumina, https://www.illumina.com) and sequenced on the Illumina NovaSeq X Plus by Novogene. De novo assembly was performed using Unicycler (v0.4.8) via the Bacterial and Viral Bioinformatics Resource Center (BV-BRC; accessed November 2025–January 2026) (*3*,*4*). Assembly quality was assessed with QUAST (v5.2.0) (*5*). Assembly completeness and contamination were additionally estimated with CheckM2 (v1.1.0) using the predict workflow on de novo assemblies (*6*). Predefined quality criteria required coverage depth of 100× or greater, CheckM2-estimated contamination less than 5%, total assembly length within the expected range for *K. pneumoniae* (4.5–6.5 Mb), fewer than 500 contigs, and N50 greater than 20 kb. Genomic characterization was performed using Pathogenwatch (accessed November 2025–January 2026) (*7*), with *Klebsiella* genomic typing based on Kleborate (v3.2.4) (*8*). For whole-genome-sequenced isolates, *K. pneumoniae* species assignment was confirmed by genomic analysis.

Core-genome single nucleotide polymorphisms (SNPs) were identified by mapping short reads to the *K. pneumoniae* NTUH-K2044 reference genome (GenBank: AP006725.1) using

Snippy (v4.6.0). A core SNP alignment was generated across all 164 isolates using snippy-core. Recombinant regions were identified and removed using Gubbins (v3.4.1) with the GTR substitution model and IQ-TREE as the tree builder (*9*,*10*). A maximum-likelihood phylogeny was inferred from the recombination-filtered SNP alignment using IQ-TREE (v3.0.1) with the GTR+Γ4 substitution model, 1000 ultrafast bootstrap replicates, and 1000 SH-aLRT replicates. The tree was visualized and annotated using iTOL v7.5 (*11*). Pairwise SNP differences were calculated from the recombination-filtered core-genome SNP alignment used for phylogenetic analysis. For each isolate pair, positions with A, C, G, or T in both isolates were compared, and the number of differing positions was counted. Positions containing ambiguous bases or gaps in either isolate were excluded from the pairwise comparison.

β-lactam resistance determinants were additionally re-evaluated in all 164 assemblies using AMRFinderPlus (v4.0.23) (*12*). We additionally screened the sequenced isolates for *peg-344* by BLASTn against de novo assemblies (*13*). The *peg-344* nucleotide sequence was used as the query, and isolates were considered positive when the best hit met ≥90% nucleotide identity and ≥90% query coverage.

Contig-level analysis of hvKp–ESBL convergence isolates

Draft genome assemblies of the three hvKp–ESBL convergence isolates were used for contig-level analysis. Contigs carrying *bla*$_{\text{CTX-M}}$ genes were identified from AMRFinderPlus outputs. Reconstructed plasmid-cluster assignment and predicted mobility were then assessed

using MOB-suite (v3.1.9) (*14*). Hypervirulence-associated loci were summarized from existing annotation outputs and interpreted in relation to the ESBL-carrying contigs.

Firth's penalized logistic regression

For the supportive analysis of 30-day mortality, we used Firth's penalized logistic regression to obtain less biased estimates under sparse outcome data (*15*). Given the small number of deaths in the hvKp group, we specified a parsimonious adjustment set (age and Charlson Comorbidity Index) to reduce overfitting and model instability in low events-per-parameter settings. Episodes with unknown 30-day vital status were treated as missing and excluded from regression models. Firth's penalized logistic regression was implemented using the R package logistf (v1.26.1).

Length of stay: additional modeling details

Episodes in which length of stay could not be ascertained were excluded from length-of-stay analyses. The log transformation ln(LOS+1) was used because length of stay included zero values. Regression coefficients were exponentiated to report adjusted ratios of (LOS+1) with 95% confidence intervals.

Total antibiotic duration: additional modeling details

Episodes with unavailable duration due to transfer, incomplete follow-up, or no treatment were treated as missing and excluded from duration analyses. To evaluate treatment burden among patients who completed antimicrobial therapy, patients who died in hospital before completion of therapy were excluded from the primary duration analysis. Sensitivity analyses repeated the adjusted models including patients who died before completion of therapy using observed duration up to death.

Prolonged therapy threshold analyses

Prolonged therapy was examined using prespecified thresholds (≥14, ≥21, and ≥28 days) as binary outcomes among treatment completers included in the primary antibiotic-duration analysis. A common base adjustment set was used, comprising age, sex, Charlson Comorbidity Index excluding age points, infection acquisition category, and primary infection source category. Because sparse outcome patterns across some infection-source strata led to separation in standard logistic regression, Firth's penalized logistic regression was used to obtain more stable and less biased estimates. In secondary threshold models, abscess complication was additionally included to assess attenuation of the association between hvKp and prolonged therapy after accounting for abscess complication.

Biomarker stratification

As a prespecified exploratory analysis, all 207 episodes were stratified by the number of PCR-positive biomarkers (0, 1–4, or 5 of 5).

Supplementary Note

Contig-level genomic context of the three hvKp–ESBL convergence isolates

Short-read assembly analysis suggested that ESBL genes and hypervirulence-associated loci were identified on separate putative plasmid-associated contigs in all three hvKp–ESBL convergence isolates, supporting independent acquisition rather than colocalization on a single reconstructed plasmid.

KML26162 (ST412/KL57) carried $bla_{\text{CTX-M-15}}$. The ESBL gene was identified on contig 37 (9.8 kb), which MOB-suite assigned to the putative plasmid-associated AA449/AI534 cluster; the corresponding reconstructed cluster was predicted to be conjugative. In contrast, *iuc*, *iro*, and *rmpA*/*rmpA2*-associated sequences were identified on separate contigs assigned to the distinct AA406/AI454 cluster, classified as non-mobilizable.

KML26192 (ST893/KL20) carried $bla_{\text{CTX-M-55}}$. The ESBL gene was identified on contig 24 (71.2 kb), assigned to the putative plasmid-associated AA277/AI090 cluster, with the corresponding reconstructed cluster predicted to be conjugative. Hypervirulence-associated

sequences were again detected on separate contigs assigned to the distinct AA406/AI454 cluster, classified as non-mobilizable.

KML26241 (ST412/KL57) carried *bla*$_{CTX\text{-}M\text{-}15}$. The ESBL gene was identified on contig 39 (9.8 kb), assigned to the putative plasmid-associated AA449/AI534 cluster, with the corresponding reconstructed cluster predicted to be conjugative. As in KML26162, hypervirulence-associated sequences were detected on separate contigs assigned to the distinct AA406/AI454 cluster, classified as non-mobilizable.

The two ST412/KL57 isolates showed closely similar contig-level contexts for *bla*$_{CTX\text{-}M\text{-}15}$ and hypervirulence-associated loci. Because these inferences were based on short-read draft assemblies, plasmid structures could not be resolved definitively.

References

1. Barrios-Camacho H, Silva-Sánchez J, Cercas-Ayala E, Lozano-Aguirre L, Duran-Bedolla J, Aguilar-Vera A, et al. PCR system for the correct differentiation of the main bacterial species of the *Klebsiella pneumoniae* complex. Arch Microbiol. 2022;204:73. PubMed https://doi.org/10.1007/s00203-021-02668-x

2. Russo TA, Olson R, Fang CT, Stoesser N, Miller M, MacDonald U, et al. Identification of biomarkers for differentiation of hypervirulent *Klebsiella pneumoniae* from classical *K. pneumoniae.* J Clin Microbiol. 2018;56:e00776-18. PubMed https://doi.org/10.1128/JCM.00776-18

3. Wick RR, Judd LM, Gorrie CL, Holt KE. Unicycler: Resolving bacterial genome assemblies from short and long sequencing reads. PLOS Comput Biol. 2017;13:e1005595. PubMed https://doi.org/10.1371/journal.pcbi.1005595

4. Shukla M, Wattam AR, Aleman A, Bhattacharya R, Bowers N, Brettin T, et al. BV-BRC: a unified bacterial and viral bioinformatics resource with expanded functionality and AI integration. Nucleic Acids Res. 2026;54(D1):D715–23. PubMed https://doi.org/10.1093/nar/gkaf1254

5. Gurevich A, Saveliev V, Vyahhi N, Tesler G. QUAST: quality assessment tool for genome assemblies. Bioinformatics. 2013;29:1072–5. PubMed https://doi.org/10.1093/bioinformatics/btt086

6. Chklovski A, Parks DH, Woodcroft BJ, Tyson GW. CheckM2: a rapid, scalable and accurate tool for assessing microbial genome quality using machine learning. Nat Methods. 2023;20:1203–12. PubMed https://doi.org/10.1038/s41592-023-01940-w

7. Argimón S, David S, Underwood A, Abrudan M, Wheeler NE, Kekre M, et al.; NIHR Global Health Research Unit on Genomic Surveillance of Antimicrobial Resistance. Rapid genomic characterization and global surveillance of *Klebsiella* using Pathogenwatch. Clin Infect Dis. 2021;73(Suppl_4):S325–35. PubMed https://doi.org/10.1093/cid/ciab784

8. Lam MMC, Wick RR, Watts SC, Cerdeira LT, Wyres KL, Holt KE. A genomic surveillance framework and genotyping tool for *Klebsiella pneumoniae* and its related species complex. Nat Commun. 2021;12:4188. PubMed https://doi.org/10.1038/s41467-021-24448-3

9. Croucher NJ, Page AJ, Connor TR, Delaney AJ, Keane JA, Bentley SD, et al. Rapid phylogenetic analysis of large samples of recombinant bacterial whole genome sequences using Gubbins. Nucleic Acids Res. 2015;43:e15. PubMed https://doi.org/10.1093/nar/gku1196

10. Wong TKF, Ly-Trong N, Ren H, Demotte P, Baños H, Roger AJ, et al. IQ-TREE 3: phylogenomic inference software using complex evolutionary models. Mol Biol Evol. 2026;43:msag117. PubMed https://doi.org/10.1093/molbev/msag117

11. Letunic I, Bork P. Interactive Tree of Life (iTOL) v6: recent updates to the phylogenetic tree display and annotation tool. Nucleic Acids Res. 2024;52(W1):W78–82. PubMed https://doi.org/10.1093/nar/gkae268

12. Feldgarden M, Brover V, Gonzalez-Escalona N, Frye JG, Haendiges J, Haft DH, et al. AMRFinderPlus and the Reference Gene Catalog facilitate examination of the genomic links among antimicrobial resistance, stress response, and virulence. Sci Rep. 2021;11:12728. PubMed https://doi.org/10.1038/s41598-021-91456-0

13. Camacho C, Coulouris G, Avagyan V, Ma N, Papadopoulos J, Bealer K, et al. BLAST+: architecture and applications. BMC Bioinformatics. 2009;10:421. PubMed https://doi.org/10.1186/1471-2105-10-421

14. Robertson J, Nash JHE. MOB-suite: software tools for clustering, reconstruction and typing of plasmids from draft assemblies. Microb Genom. 2018;4:e000206. PubMed https://doi.org/10.1099/mgen.0.000206

15. Firth D. Bias reduction of maximum likelihood estimates. Biometrika. 1993;80:27–38.

https://doi.org/10.1093/biomet/80.1.27

Appendix Table 1. Primers and PCR conditions used for detection of the 5 biomarkers

Biomarker	Primer name	Sequence (5′–3′)	Annealing temperature	Extension time	Expected product size
rmpA	Forward	GAGTAGTTAATAA ATCAATAGCAAT	50°C	40 s	332 bp
	Reverse	CAGTAGGCATTG CAGCA			
rmpA2	Forward	GTGCAATAAGGAT GTTACATTA	50°C	40 s	430 bp
	Reverse	GGATGCCCTCCT CCTG			
iucA	F2	GCTTATTTCTCCC CAACCC	59°C	50 s	583 bp
	R2	TCAGCCCTTTAGC GACAAG			
iroB	F1	ATCTCATCATCTA CCCTCCGCTC	59°C	30 s	235 bp

Biomarker	Primer name	Sequence (5′–3′)	Annealing temperature	Extension time	Expected product size
	R1	GGTTCGCCGTCG TTTTCAA			
peg-344	F2	AAAGGACAGAAA GCCAGTG	53°C	30 s	411 bp
	R2	CAATGACGAGGG GGATAATC			

Appendix Table 2. Full Baseline Characteristics, Comorbidity Details, Antimicrobial Regimens, Laboratory Variables, and Outcomes by Hypervirulent Status

Characteristic or Condition	Episodes, No. (%)		Available (n/N)	p value
	hvKp (n = 28)	cKp (n = 179)		
Comorbidities				
Charlson Comorbidity Index score ≥2	16 (57)	143 (80)	207/207	0.01
Chronic pulmonary disease	2 (7)	18 (10)	207/207	>0.99
Congestive heart failure	5 (18)	37 (21)	207/207	>0.99
Connective tissue disease	2 (7)	13 (7)	207/207	>0.99
Cerebrovascular disease/ transient ischemic attack	7 (25)	26 (15)	207/207	0.17

	Episodes, No. (%)			
Characteristic or Condition	hvKp (n = 28)	cKp (n = 179)	Available (n/N)	p value
Dementia	6 (21)	18 (10)	207/207	0.11
Diabetes (no complications)	8 (29)	46 (26)	207/207	0.82
Diabetes with complications	1 (4)	16 (9)	207/207	0.48
Hemiplegia	2 (7)	6 (3)	207/207	0.30
Leukemia	0 (0.0)	9 (5)	207/207	0.61
Mild liver disease	1 (4)	5 (3)	207/207	0.59
Moderate/severe liver disease	0 (0.0)	4 (2)	207/207	>0.99
Lymphoma	0 (0.0)	23 (13)	207/207	0.04
Moderate/severe chronic kidney disease	3 (11)	23 (13)	207/207	>0.99
Myocardial infarction	4 (14)	7 (4)	207/207	0.04
Peptic ulcer disease	1 (4)	24 (13)	207/207	0.21
Peripheral vascular disease	2 (7)	14 (8)	207/207	>0.99
Localized solid tumor	5 (18)	41 (23)	207/207	0.63
Metastatic solid tumor	3 (11)	23 (13)	207/207	>0.99
Immunosuppression				
Febrile neutropenia	1 (4)	13 (7)	207/207	0.70
Chemotherapy	3 (11)	51 (28)	207/207	0.06
Systemic corticosteroids	3 (11)	39 (22)	207/207	0.21

	Episodes, No. (%)			
Characteristic or Condition	hvKp (n = 28)	cKp (n = 179)	Available (n/N)	p value
Immunosuppressant use	0 (0.0)	9 (5)	207/207	0.61
History of transplantation	0 (0.0)	2 (1)	207/207	>0.99
HIV infection	0 (0.0)	0 (0.0)	207/207	NA
Laboratory values				
Leukocyte count, $10^2/\mu L$	145 (97, 164)	101 (50, 151)	207/207	0.02
Infection characteristics				
Any abscess	17 (61)	23 (13)	207/207	<0.001
Liver abscess	9 (32)	12 (7)	207/207	<0.001
Other intra-abdominal and pelvic abscesses	3 (11)	10 (6)	207/207	0.39
Lung abscess/necrotizing pneumonia	2 (7)	3 (2)	207/207	0.14
Urogenital/prostatic abscess	4 (14)	0 (0.0)	207/207	<0.001
Ocular sites	1 (4)	0 (0.0)	207/207	0.14
Central nervous system abscess	2 (7)	0 (0.0)	207/207	0.01
Musculoskeletal/soft tissue infection	2 (7)	0 (0.0)	207/207	0.01
Antimicrobial therapy				
Empiric therapy (class)				0.69
3rd-generation cephalosporin	6 (21)	35 (20)	207/207	
4th-generation cephalosporin	1 (4)	4 (2)	207/207	

	Episodes, No. (%)			
Characteristic or Condition	hvKp (n = 28)	cKp (n = 179)	Available (n/N)	p value
Other cephalosporins/penicillins	1 (4)	9 (5)	207/207	
β-lactam/β-lactamase inhibitor	18 (64)	124 (69)	207/207	
Carbapenem	2 (7)	5 (3)	207/207	
Oral agents	0 (0.0)	1 (<1)	207/207	
No empiric therapy	0 (0.0)	1 (<1)	207/207	
Appropriate empiric therapy	27 (96)	151 (84)	207/207	0.14
Definitive therapy (class)				<0.001
3rd-generation cephalosporin	11 (39)	11 (6)	207/207	
4th-generation cephalosporin	1 (4)	3 (2)	207/207	
Other cephalosporins/penicillins	6 (21)	65 (36)	207/207	
β-lactam/β-lactamase inhibitor	5 (18)	71 (40)	207/207	
Carbapenem	2 (7)	13 (7)	207/207	
Oral agents	3 (11)	9 (5)	207/207	
Other	0 (0.0)	1 (<1)	207/207	
No definitive therapy	0 (0.0)	6 (3)	207/207	
Treatment				
Source control (surgery/drainage)	10 (36)	52 (29)	207/207	0.51
Severity/organ support				

Characteristic or Condition	Episodes, No. (%)		Available (n/N)	p value
	hvKp (n = 28)	cKp (n = 179)		
Intensive care unit admission	5 (18)	25 (14)	207/207	0.57
Vasopressor use (proxy for shock)	7 (25)	51 (28)	207/207	0.82
Mechanical ventilation	4 (14)	5 (3)	207/207	0.02
Outcomes				
14-d mortality	3 (11)	24 (14)	202/207	>0.99
30-d mortality	4 (15)	34 (20)	197/207	0.61
90-d mortality	5 (22)	56 (35)	183/207	0.24
In-hospital mortality	3 (11)	34 (19)	207/207	0.43

Data are n (%) or median (IQR), unless otherwise indicated. P values were calculated using Fisher exact test for categorical variables and the Mann-Whitney U test for continuous variables and are provided for descriptive purposes. Available (n/N) indicates the number of episodes with available data out of the total in each group; percentages were calculated using available cases as the denominator. Appropriate empiric therapy was defined as initial empiric antimicrobial therapy with in vitro activity against the causative isolate.

Abbreviations: cKp, classical *K. pneumoniae*; HIV, human immunodeficiency virus; hvKp, hypervirulent *K. pneumoniae*; IQR, interquartile range; NA, not applicable.

Appendix Table 3. Antimicrobial Non-susceptibility by Category and Multidrug-Resistant Status

Antimicrobial category	Agents tested	hvKp (n = 28)	cKp (n = 179)	p value
Penicillins	Piperacillin	7 (25)	66 (37)	0.29
Penicillins + β-lactamase inhibitors	Ampicillin–sulbactam, amoxicillin–clavulanate	3 (11)	47 (26)	0.10
Antipseudomonal penicillins + β-lactamase inhibitors	Piperacillin–tazobactam	1 (4)	15 (8)	0.70
Non-extended-spectrum cephalosporins (1st/2nd gen)	Cefazolin	4 (14)	42 (23)	0.34
Extended-spectrum cephalosporins (3rd/4th gen)	Ceftriaxone, cefepime	3 (11)	28 (16)	0.78
Cephamycins	Cefmetazole	0 (0)	1 (1)	>0.99
Carbapenems	Imipenem, meropenem	0 (0)	1 (1)	>0.99
Monobactams	Aztreonam	3 (11)	22 (12)	>0.99
Aminoglycosides	Gentamicin, tobramycin, amikacin	2 (7)	24 (13)	0.54
Fluoroquinolones	Levofloxacin, ciprofloxacin	3 (11)	35 (20)	0.43
Tetracyclines	Minocycline	0 (0)	30 (17)	0.02
Folate pathway antagonists	Trimethoprim–sulfamethoxazole	3 (11)	46 (26)	0.10
Multidrug resistance	≥3 categories	3 (11)	53 (30)	0.04

Antimicrobial category	Agents tested	hvKp (n = 28)	cKp (n = 179)	p value

Data are n (%). P values were calculated using Fisher exact test and are provided for descriptive purposes. Abbreviations: cKp, classical *K. pneumoniae*; hvKp, hypervirulent *K. pneumoniae*.

Appendix Table 4. Missingness and Analysis Populations

	Available No.		Missing No.		
Variable or outcome	hvKp (n = 28)	cKp (n = 179)	hvKp (n = 28)	cKp (n = 179)	Included in main model
Abscess complication	28 (100)	179 (100)	0 (0)	0 (0)	Yes
Length of stay	28 (100)	178 (99)	0 (0)	1 (<1)	Yes
Total antibiotic duration	24 (86)	144 (80)	4 (14)	35 (20)	Yes, among treatment completers
30-d mortality	27 (96)	170 (95)	1 (4)	9 (5)	Supportive
Age	28 (100)	179 (100)	0 (0)	0 (0)	Yes
Sex	28 (100)	179 (100)	0 (0)	0 (0)	Yes
Charlson Comorbidity Index	28 (100)	179 (100)	0 (0)	0 (0)	Yes
Acquisition category	28 (100)	179 (100)	0 (0)	0 (0)	Yes
Primary source category	28 (100)	179 (100)	0 (0)	0 (0)	Yes

Data are n (%). For total antibiotic duration, available no refers to the primary analysis population among treatment completers. Thirty-nine episodes were excluded from this analysis, including 37 that ended in death before completion of therapy and 2 with unavailable duration data.

Appendix Table 5. Multivariable Logistic Regression for Abscess Complication

Covariate	Adjusted odds ratio	95% CI	p value
hvKp (vs cKp)	10.7	4.36–26.21	<0.001
Age	1.00	0.97–1.03	0.94
Male sex (vs female)	0.76	0.34–1.69	0.50
Charlson Comorbidity Index	0.91	0.77–1.07	0.26

Adjusted odds ratios are presented; odds ratios for age and Charlson Comorbidity Index are per 1-unit increase. Analyses used complete cases (n = 207). Abbreviations: cKp, classical *K. pneumoniae*; hvKp, hypervirulent *K. pneumoniae*.

Appendix Table 6. Clinical Outcomes Stratified by Number of PCR-Positive Biomarkers Among 207 *Klebsiella pneumoniae* Bloodstream Infection Episodes

Covariate	0 markers (n = 171)	1–4 markers (n = 8)	5 markers (n = 28)	Available (n/N)
Abscess complication	21/171 (12)	2/8 (25)	17/28 (61)	207/207
Metastatic infection	1/171 (0.6)	0/8 (0.0)	4/28 (14)	207/207

Covariate	0 markers (n = 171)	1–4 markers (n = 8)	5 markers (n = 28)	Available (n/N)
14-d mortality	23/167 (14)	1/8 (13)	3/27 (11)	202/207
30-d mortality	33/162 (20)	1/8 (13)	4/27 (15)	197/207
90-d mortality	54/153 (35)	2/7 (29)	5/23 (22)	183/207
Intensive care unit admission	24/171 (14)	1/8 (13)	5/28 (18)	207/207
Vasopressor use	49/171 (29)	2/8 (25)	7/28 (25)	207/207
Mechanical ventilation	5/171 (3)	0/8 (0.0)	4/28 (14)	207/207
Length of stay, days, median (IQR)	14 (11, 18)	12 (10, 19)	28 (14, 42)	206/207
Antibiotic duration, days, median (IQR)	14 (14, 18)	14 (14, 15)	43 (16, 67)	168/207

Data are n (%) or median (IQR), unless otherwise indicated. Biomarkers comprise *rmpA*, *rmpA2*, *iucA*, *iroB*, and *peg-344*, detected by PCR. All 8 isolates in the 1–4-marker group carried 3 of 5 biomarkers (*rmpA*, *iroB*, and *peg-344* positive; *iucA* and *rmpA2* negative). Denominators vary because of missing vital status at 14, 30, and 90 d. Abbreviations: IQR, interquartile range.

Appendix Table 7. Multivariable Linear Regression for Post-Onset Length of Stay

Covariate	Model A adjusted ratio (95% CI)	Model A, *P value*	Model B adjusted ratio (95% CI)	Model B *P value*
hvKp (vs cKp)	1.60 (1.18–2.16)	0.002	1.07 (0.79–1.45)	0.65
Abscess complication (yes vs no)	Not applicable	Not applicable	2.35 (1.80–3.06)	<0.001
Age	1.00 (0.99–1.00)	0.23	1.00 (0.99–1.00)	0.21
Male sex (vs female)	1.10 (0.89–1.36)	0.39	1.13 (0.93–1.37)	0.24
Charlson Comorbidity Index	0.99 (0.94–1.03)	0.53	0.99 (0.95–1.03)	0.73
Community-acquired (vs healthcare-associated)	1.04 (0.84–1.30)	0.70	0.95 (0.78–1.16)	0.61

Outcome was ln(length of stay + 1). Coefficients were exponentiated to report adjusted ratios of (length of stay + 1). Model A adjusted for age, sex, Charlson Comorbidity Index excluding age points, and acquisition category. Model B included all covariates in Model A plus any abscess complication. Analyses used complete cases (n = 206).

Abbreviations: CI, confidence interval; cKp, classical *K. pneumoniae*; hvKp, hypervirulent *K. pneumoniae*.

Appendix Table 8. Multivariable Log-Linear Regression for Total Antibiotic Duration Among Treatment Completers

Covariate	Model A adjusted ratio (95% CI)	Model A, P value	Model B adjusted ratio (95% CI)	Model B P value
hvKp (vs cKp)	2.13 (1.64–2.77)	<0.001	1.24 (1.00–1.54)	0.05
Abscess complication (yes vs no)	Not applicable	Not applicable	3.08 (2.53–3.76)	<0.001
Age	1.00 (0.99–1.00)	0.54	1.00 (0.99–1.00)	0.53
Male sex (vs female)	0.93 (0.77–1.13)	0.46	0.97 (0.84–1.12)	0.70
Charlson Comorbidity Index	0.99 (0.95–1.03)	0.70	1.00 (0.97–1.04)	0.76
Community-acquired (vs healthcare-associated)	1.04 (0.85–1.26)	0.72	0.92 (0.80–1.07)	0.28
Primary infection source: catheter-related	1.34 (0.71–2.53)	0.37	1.38 (0.86–2.22)	0.18
Primary infection source: other	2.09 (0.90–4.83)	0.08	1.00 (0.53–1.89)	0.99
Primary infection source: respiratory	0.91 (0.56–1.48)	0.70	0.87 (0.60–1.24)	0.43
Primary infection source: unknown	1.17 (0.88–1.56)	0.29	1.16 (0.94–1.44)	0.17
Primary infection source: intra-abdominal	1.28 (1.02–1.61)	0.03	1.15 (0.97–1.37)	0.11

Outcome was ln(total antibiotic duration). Coefficients were exponentiated to report adjusted ratios; ratios greater than 1 indicate longer duration.

Model A adjusted for age, sex, Charlson Comorbidity Index, infection acquisition category, and primary infection source category. Model B included all covariates in Model A plus abscess complication. Analyses were restricted to treatment completers and used complete cases (n = 168).

Abbreviations: CI, confidence interval; cKp, classical *K. pneumoniae*; hvKp, hypervirulent *K. pneumoniae*.

Appendix Table 9. Firth's Penalized Logistic Regression for Prolonged Antibiotic Therapy

Threshold	Model	Covariate	Adjusted odds ratio (95% CI)	*P value*
≥14 d	Base model	hvKp	2.21 (0.62–11.79)	0.24
≥14 d	+Abs model	hvKp	0.65 (0.14–4.15)	0.62
≥14 d	+Abs model	Abscess	23.64 (2.58–3226.46)	0.001
≥21 d	Base model	hvKp	6.85 (2.58–19.93)	<0.001
≥21 d	+Abs model	hvKp	1.38 (0.20–7.35)	0.72
≥21 d	+Abs model	Abscess	143.69 (25.84–2042.25)	<0.001
≥28 d	Base model	hvKp	6.71 (2.51–19.43)	<0.001
≥28 d	+Abs model	hvKp	1.30 (0.23–6.34)	0.75
≥28 d	+Abs model	Abscess	59.26 (15.59–354.11)	<0.001

Prolonged therapy was defined using prespecified thresholds of ≥14, ≥21, and ≥28 d. Base covariates were age, sex, Charlson Comorbidity Index, infection acquisition category, and primary infection source category. The +Abs model included all base covariates plus abscess complication. Analyses were restricted to treatment completers and used complete cases (n = 168). Abbreviations: CI, confidence interval; hvKp, hypervirulent *K. pneumoniae*.

Appendix Table 10. Firth's Penalized Logistic Regression for 30-Day Mortality

Covariate	Adjusted odds ratio (95% CI)	p value
hvKp (vs cKp)	0.85 (0.25–2.39)	0.77
Age	1.03 (1.00–1.06)	0.07
Charlson Comorbidity Index	1.25 (1.08–1.45)	0.003

Adjusted odds ratios are presented; odds ratios for age and Charlson Comorbidity Index are per 1-unit increase. The model adjusted for age and Charlson Comorbidity Index and used complete cases with available 30-d vital status (n = 197). Abbreviations: CI, confidence interval; cKp, classical *K. pneumoniae*; hvKp, hypervirulent *K. pneumoniae*.

Appendix Table 11. Clinical Characteristics of Randomly Sampled Classical *Klebsiella pneumoniae* Isolates and Those Not Included in the Random Sample

Characteristic	Randomly sampled cKp isolates (n = 125)	cKp isolates not included in the random sample (n = 54)	P value
Age, years, median (IQR)	79 (70, 86)	76 (65, 84)	0.22
Male sex	74 (59)	34 (63)	0.74
Charlson Comorbidity Index, median (IQR)	3 (2, 5)	3 (2, 5)	0.90

Characteristic	Randomly sampled cKp isolates (n = 125)	cKp isolates not included in the random sample (n = 54)	P value
Onset classification			0.13
Healthcare-associated	75 (60)	39 (72)	0.13
Community-acquired	50 (40)	15 (28)	
Primary source of infection			0.01
Urinary tract	25 (20)	12 (22)	
Catheter-related	0 (0.0)	5 (9)	
Intra-abdominal	61 (49)	22 (41)	
Respiratory	4 (3)	1 (2)	
Other	0 (0.0)	1 (2)	
Unknown	35 (28)	13 (24)	
Any abscess	15 (12)	8 (15)	0.63
30-d mortality	22 (18)	12 (25)	0.40
Length of stay, days, median (IQR)	14 (12, 18)	14 (10, 18)	0.76
Third-generation cephalosporin-resistant	20 (16)	8 (15)	>0.99

Data are n (%) or median (IQR), unless otherwise indicated. P values were calculated using Fisher exact test for categorical variables and the Mann-Whitney U test for continuous variables and are provided for descriptive purposes. The between-group difference in primary infection source reflected catheter-related infections being present only in the group not included in the random sample (0/125 vs 5/54).

Abbreviations: cKp, classical *K. pneumoniae*; IQR, interquartile range; WGS, whole-genome sequencing.

Appendix Table 12. Isolate-Level Genomic and Clinical Summary of Whole-Genome-Sequenced Isolates

Isolate ID	Classification	Detected biomarkers	ST/KL	Kleborate virulence score	CTX-M gene	AMR phenotype	Abscess complication	WGS selection category
KML26001	cKp	none	ST45/KL24	1	$bla_{CTX\text{-}M\text{-}15}$	3GCR	No	Random cKp
KML26004	cKp	none	ST4087/KL58	0	none	non-3GCR	No	Random cKp
KML26006	cKp	none	ST626/KL54	0	none	non-3GCR	No	Random cKp
KML26007	hvKp	all 5	ST86/KL2	4	none	non-3GCR	Yes	hvKp
KML26009	hvKp	all 5	ST23/KL1	5	none	non-3GCR	Yes	hvKp
KML26010	cKp	none	ST307/KL102	0	$bla_{CTX\text{-}M\text{-}15}$	3GCR	No	Added cKp
KML26012	cKp	none	ST1334/KL125	0	none	non-3GCR	No	Random cKp
KML26014	cKp	none	ST292/KL139	0	none	non-3GCR	No	Random cKp
KML26016	cKp	none	ST200/KL15	0	none	non-3GCR	No	Random cKp

Isolate ID	Classification	Detected biomarkers	ST/KL	Kleborate virulence score	CTX-M gene	AMR phenotype	Abscess complication	WGS selection category
KML26019	hvKp	all 5	ST86/KL2	4	none	non-3GCR	No	hvKp
KML26020	cKp	none	ST37/KL15	0	none	non-3GCR	No	Random cKp
KML26021	cKp	none	ST37/KL7	0	none	non-3GCR	No	Random cKp
KML26022	cKp	none	ST1087/KL132	0	none	non-3GCR	No	Random cKp
KML26023	cKp	*rmpA*, *iroB*, *peg-344*	ST268/KL20	2	none	non-3GCR	No	Random cKp
KML26025	cKp	none	ST1728/KL116	0	none	non-3GCR	No	Random cKp
KML26028	cKp	*rmpA*, *iroB*, *peg-344*	ST39/KL23	0	none	non-3GCR	No	Random cKp
KML26031	cKp	none	ST37/KL136	0	none	non-3GCR	No	Random cKp
KML26032	hvKp	all 5	ST268/KL20	5	none	non-3GCR	No	hvKp

Isolate ID	Classification	Detected biomarkers	ST/KL	Kleborate virulence score	CTX-M gene	AMR phenotype	Abscess complication	WGS selection category
KML26033	cKp	none	ST6/KL7	1	none	non-3GCR	No	Random cKp
KML26035	cKp	none	ST277/KL46	0	none	non-3GCR	No	Random cKp
KML26037	hvKp	all 5	ST23/KL1	5	none	non-3GCR	Yes	hvKp
KML26039	cKp	none	ST4568/KL127	0	none	non-3GCR	No	Random cKp
KML26040	cKp	none	ST1537/KL24	0	none	non-3GCR	No	Random cKp
KML26042	cKp	none	ST45/KL24	1	none	non-3GCR	No	Random cKp
KML26043	cKp	none	ST629/KL10	0	none	non-3GCR	No	Random cKp
KML26044	hvKp	all 5	ST65/KL2	5	none	non-3GCR	No	hvKp
KML26047	cKp	none	ST307/KL102	0	$bla_{CTX\text{-}M\text{-}15}$	3GCR	No	Added cKp

Isolate ID	Classification	Detected biomarkers	ST/KL	Kleborate virulence score	CTX-M gene	AMR phenotype	Abscess complication	WGS selection category
KML26048	cKp	none	ST36/KL27	0	none	non-3GCR	Yes	Random cKp
KML26049	hvKp	all 5	ST65/KL2	3	none	non-3GCR	Yes	hvKp
KML26052	cKp	none	ST280/KL23	0	none	non-3GCR	No	Random cKp
KML26054	cKp	none	ST4422/KL52	0	none	non-3GCR	Yes	Random cKp
KML26055	cKp	none	ST461/KL64	0	none	non-3GCR	No	Random cKp
KML26064	cKp	none	ST37/KL15	0	*bla*$_{CTX-M-14}$	3GCR	No	Random cKp
KML26065	cKp	none	ST2028/KL62	0	none	non-3GCR	No	Random cKp
KML26066	hvKp	all 5	ST65/KL2	5	none	non-3GCR	Yes	hvKp
KML26067	cKp	none	ST942/KL21	0	none	non-3GCR	No	Random cKp

Isolate ID	Classification	Detected biomarkers	ST/KL	Kleborate virulence score	CTX-M gene	AMR phenotype	Abscess complication	WGS selection category
KML26069	cKp	none	ST12/KL122	0	none	non-3GCR	No	Random cKp
KML26070	cKp	none	ST784/KL146	0	none	non-3GCR	Yes	Random cKp
KML26073	cKp	none	ST464/KL34	0	none	non-3GCR	No	Random cKp
KML26074	cKp	none	ST20/KL28	0	none	non-3GCR	Yes	Random cKp
KML26075	cKp	none	ST45/KL43	1	none	non-3GCR	No	Random cKp
KML26076	cKp	none	ST37/KL15	0	*bla*$_{CTX-M-14}$	3GCR	No	Random cKp
KML26077	cKp	none	ST37/KL15	0	*bla*$_{CTX-M-14}$	3GCR	No	Random cKp
KML26078	cKp	none	ST307/KL102	0	*bla*$_{CTX-M-15}$	3GCR	No	Random cKp

Isolate ID	Classification	Detected biomarkers	ST/KL	Kleborate virulence score	CTX-M gene	AMR phenotype	Abscess complication	WGS selection category
KML26079	cKp	none	ST111/KL63	1	none	non-3GCR	No	Random cKp
KML26080	cKp	none	ST200/KL114	0	none	non-3GCR	No	Random cKp
KML26084	hvKp	all 5	ST23/KL1	5	none	non-3GCR	Yes	hvKp
KML26089	cKp	none	ST5011/KL147	0	none	non-3GCR	No	Random cKp
KML26090	cKp	*rmpA*, *iroB*, *peg-344*	ST39/KL23	0	none	non-3GCR	No	Random cKp
KML26091	cKp	none	ST219/KL121	0	none	non-3GCR	No	Random cKp
KML26094	cKp	none	ST37/KL15	0	$bla_{CTX\text{-}M\text{-}14}$	3GCR	No	Random cKp
KML26095	cKp	none	ST200/KL22	0	none	non-3GCR	Yes	Random cKp
KML26097	cKp	*rmpA*, *iroB*, *peg-344*	ST23/KL1	2	none	non-3GCR	Yes	Random cKp

Isolate ID	Classification	Detected biomarkers	ST/KL	Kleborate virulence score	CTX-M gene	AMR phenotype	Abscess complication	WGS selection category
KML26099	hvKp	all 5	ST29/KL54	4	none	non-3GCR	No	hvKp
KML26101	hvKp	all 5	ST23/KL1	5	none	non-3GCR	Yes	hvKp
KML26102	cKp	none	ST37/KL15	0	*bla*$_{CTX-M-14}$	3GCR	No	Added cKp
KML26103	hvKp	all 5	ST23/KL1	5	none	non-3GCR	No	hvKp
KML26104	cKp	none	ST1996/KL128	0	none	non-3GCR	No	Random cKp
KML26105	cKp	none	ST4081/KL125	0	none	non-3GCR	No	Random cKp
KML26107	cKp	none	unknown/KL12	0	none	non-3GCR	No	Random cKp
KML26108	hvKp	all 5	ST23/KL1	5	none	non-3GCR	Yes	hvKp
KML26109	hvKp	all 5	ST23/KL1	5	none	non-3GCR	Yes	hvKp
KML26112	cKp	none	ST14/KL2	0	*bla*$_{CTX-M-15}$	3GCR	No	Random cKp
KML26113	cKp	none	ST7874/KL38	0	none	non-3GCR	No	Random cKp

Isolate ID	Classification	Detected biomarkers	ST/KL	Kleborate virulence score	CTX-M gene	AMR phenotype	Abscess complication	WGS selection category
KML26114	cKp	none	ST12/KL122	0	none	non-3GCR	No	Random cKp
KML26115	hvKp	all 5	ST65/KL2	3	none	non-3GCR	Yes	hvKp
KML26116	cKp	none	ST252/KL51	0	none	non-3GCR	No	Random cKp
KML26117	cKp	none	ST45/KL5	1	*bla*$_{CTX-M-27}$	3GCR	No	Random cKp
KML26120	cKp	none	ST133/KL116	2	none	non-3GCR	Yes	Random cKp
KML26121	hvKp	all 5	ST65/KL2	3	none	non-3GCR	Yes	hvKp
KML26122	hvKp	all 5	ST65/KL2	3	none	non-3GCR	Yes	hvKp
KML26125	cKp	none	ST1017/KL58	0	none	non-3GCR	No	Random cKp
KML26126	cKp	none	ST2657/KL31	0	none	non-3GCR	No	Random cKp
KML26127	cKp	none	ST25/KL2	1	none	non-3GCR	No	Random cKp

Isolate ID	Classification	Detected biomarkers	ST/KL	Kleborate virulence score	CTX-M gene	AMR phenotype	Abscess complication	WGS selection category
KML26128	cKp	none	ST39/KL23	4	none	non-3GCR	No	Random cKp
KML26130	cKp	none	ST45/KL43	1	none	non-3GCR	No	Random cKp
KML26131	cKp	none	ST37/KL38	0	none	non-3GCR	No	Random cKp
KML26132	cKp	none	ST37/KL15	0	*bla*$_{CTX-M-14}$	3GCR	No	Random cKp
KML26134	hvKp	all 5	ST412/KL57	3	none	non-3GCR	No	hvKp
KML26136	cKp	none	ST1948/KL24	0	none	non-3GCR	No	Random cKp
KML26137	cKp	none	ST45/KL43	4	none	non-3GCR	Yes	Random cKp
KML26138	cKp	none	ST45/KL43	1	none	non-3GCR	No	Random cKp
KML26142	hvKp	all 5	ST23/KL1	5	none	non-3GCR	Yes	hvKp

Isolate ID	Classification	Detected biomarkers	ST/KL	Kleborate virulence score	CTX-M gene	AMR phenotype	Abscess complication	WGS selection category
KML26146	cKp	none	ST15/KL24	1	none	non-3GCR	No	Random cKp
KML26152	cKp	none	ST556/KL62	0	none	non-3GCR	No	Random cKp
KML26154	cKp	none	ST1145/KL30	0	none	non-3GCR	No	Random cKp
KML26155	cKp	none	ST1145/KL30	1	none	non-3GCR	No	Random cKp
KML26156	cKp	none	ST876/KL10	0	none	non-3GCR	No	Random cKp
KML26157	cKp	none	ST307/KL102	0	$bla_{CTX-M-15}$	3GCR	Yes	Added cKp
KML26160	cKp	*rmpA*, *iroB*, *peg-344*	ST592/KL57	2	none	non-3GCR	No	Added cKp
KML26162	hvKp	all 5	ST412/KL57	4	$bla_{CTX-M-15}$	3GCR	No	hvKp
KML26165	cKp	none	ST307/KL102	0	$bla_{CTX-M-15}$	3GCR	No	Random cKp

Isolate ID	Classification	Detected biomarkers	ST/KL	Kleborate virulence score	CTX-M gene	AMR phenotype	Abscess complication	WGS selection category
KML26167	cKp	none	ST17/KL23	0	none	non-3GCR	No	Random cKp
KML26172	cKp	none	ST323/KL21	0	none	non-3GCR	No	Random cKp
KML26173	cKp	none	ST22/KL9	0	none	non-3GCR	Yes	Random cKp
KML26174	cKp	none	ST307/KL102	0	*bla*$_{CTX-M-15}$	3GCR	No	Random cKp
KML26175	cKp	none	ST12/KL122	0	none	non-3GCR	No	Random cKp
KML26176	cKp	none	ST35/KL16	4	none	non-3GCR	No	Random cKp
KML26179	cKp	none	ST200/KL27	0	none	non-3GCR	No	Random cKp
KML26183	cKp	none	ST76/KL35	0	none	non-3GCR	No	Random cKp

Isolate ID	Classification	Detected biomarkers	ST/KL	Kleborate virulence score	CTX-M gene	AMR phenotype	Abscess complication	WGS selection category
KML26186	cKp	none	unknown/KL34	0	none	non-3GCR	No	Random cKp
KML26187	hvKp	all 5	ST65/KL2	3	none	non-3GCR	No	hvKp
KML26189	cKp	none	ST337/KL174	0	none	non-3GCR	No	Random cKp
KML26190	cKp	none	ST1537/KL24	0	none	non-3GCR	No	Random cKp
KML26192	hvKp	all 5	ST893/KL20	4	*bla*$_{CTX-M-55}$	3GCR	Yes	hvKp
KML26193	cKp	none	ST353/KL110	1	*bla*$_{CTX-M-15}$	3GCR	No	Random cKp
KML26196	cKp	none	ST5240/KL102	0	none	non-3GCR	Yes	Random cKp
KML26198	cKp	none	ST307/KL102	1	*bla*$_{CTX-M-15}$	3GCR	No	Random cKp
KML26199	cKp	none	ST200/KL35	0	none	non-3GCR	No	Random cKp

Isolate ID	Classification	Detected biomarkers	ST/KL	Kleborate virulence score	CTX-M gene	AMR phenotype	Abscess complication	WGS selection category
KML26202	cKp	none	ST22/KL9	0	none	non-3GCR	No	Random cKp
KML26203	hvKp	all 5	a single-locus variant of ST23 /KL1	5	none	non-3GCR	No	hvKp
KML26205	cKp	*rmpA*, *iroB*, *peg-344*	ST65/KL2	2	none	non-3GCR	No	Added cKp
KML26207	hvKp	all 5	ST23/KL1	5	none	non-3GCR	Yes	hvKp
KML26209	cKp	none	ST133/KL23	0	none	non-3GCR	No	Random cKp
KML26210	cKp	none	ST307/KL102	0	$bla_{CTX\text{-}M\text{-}15}$	3GCR	No	Random cKp
KML26212	cKp	none	ST37/KL15	0	$bla_{CTX\text{-}M\text{-}14}$	3GCR	No	Random cKp
KML26213	cKp	*rmpA*, *iroB*, *peg-344*	ST111/KL63	1	none	non-3GCR	No	Random cKp

Isolate ID	Classification	Detected biomarkers	ST/KL	Kleborate virulence score	CTX-M gene	AMR phenotype	Abscess complication	WGS selection category
KML26217	cKp	none	ST540/KL64	0	none	non-3GCR	No	Random cKp
KML26219	cKp	none	ST461/KL62	0	none	non-3GCR	No	Random cKp
KML26221	cKp	none	ST3369/KL116	0	none	non-3GCR	No	Random cKp
KML26222	hvKp	all 5	ST65/KL2	5	none	non-3GCR	No	hvKp
KML26226	cKp	none	ST353/KL110	1	*bla*$_{CTX\text{-}M\text{-}15}$	3GCR	Yes	Added cKp
KML26229	cKp	none	ST20/KL28	0	none	non-3GCR	No	Random cKp
KML26231	cKp	none	ST730/KL51	0	none	non-3GCR	No	Random cKp
KML26232	cKp	none	ST45/KL24	1	none	non-3GCR	No	Random cKp
KML26233	cKp	none	ST611/KL151	0	none	non-3GCR	Yes	Random cKp

Isolate ID	Classification	Detected biomarkers	ST/KL	Kleborate virulence score	CTX-M gene	AMR phenotype	Abscess complication	WGS selection category
KML26235	cKp	none	ST22/KL9	0	none	non-3GCR	No	Random cKp
KML26236	cKp	none	ST5405/KL127	0	none	non-3GCR	No	Random cKp
KML26238	cKp	none	ST353/KL110	1	*bla*$_{CTX-M}$ family	3GCR	Yes	Added cKp
KML26239	cKp	none	ST2158/KL25	0	none	non-3GCR	No	Random cKp
KML26240	cKp	none	ST1537/KL137	0	none	non-3GCR	Yes	Random cKp
KML26241	hvKp	all 5	ST412/KL57	4	*bla*$_{CTX-M-15}$	3GCR	No	hvKp
KML26242	cKp	none	ST2123/KL28	0	none	non-3GCR	No	Random cKp
KML26243	cKp	none	ST39/KL23	0	none	non-3GCR	Yes	Random cKp
KML26244	cKp	none	unknown/KL15	0	none	non-3GCR	No	Random cKp

Isolate ID	Classification	Detected biomarkers	ST/KL	Kleborate virulence score	CTX-M gene	AMR phenotype	Abscess complication	WGS selection category
KML26245	cKp	none	ST22/KL9	0	none	non-3GCR	No	Random cKp
KML26246	cKp	none	ST22/KL9	0	none	non-3GCR	No	Random cKp
KML26247	cKp	none	ST469/KL139	0	*bla*$_{CTX-M-15}$	3GCR	No	Random cKp
KML26248	cKp	none	ST469/KL139	0	*bla*$_{CTX-M-15}$	3GCR	No	Added cKp
KML26249	cKp	none	ST485/KL28	0	none	non-3GCR	No	Random cKp
KML26254	cKp	none	ST147/KL31	2	*bla*$_{CTX-M-15}$	3GCR	No	Random cKp
KML26255	hvKp	all 5	ST412/KL57	3	none	non-3GCR	Yes	hvKp
KML26259	cKp	none	unknown/KL126	0	none	non-3GCR	No	Random cKp
KML26260	cKp	none	ST461/KL51	0	none	non-3GCR	No	Random cKp

Isolate ID	Classification	Detected biomarkers	ST/KL	Kleborate virulence score	CTX-M gene	AMR phenotype	Abscess complication	WGS selection category
KML26261	cKp	none	ST2854/KL183	0	none	non-3GCR	No	Random cKp
KML26262	cKp	none	ST867/KL184	0	none	non-3GCR	No	Random cKp
KML26264	cKp	none	ST353/KL110	1	*bla*$_{CTX-M}$ family	3GCR	No	Random cKp
KML26265	cKp	none	ST3811/KL52	0	none	non-3GCR	Yes	Random cKp
KML26267	cKp	none	ST17/KL2	0	none	non-3GCR	No	Random cKp
KML26268	cKp	none	ST1526/KL55	1	none	non-3GCR	Yes	Random cKp
KML26270	cKp	none	ST353/KL110	1	*bla*$_{CTX-M}$ family	3GCR	No	Random cKp
KML26273	cKp	none	ST661/KL140	0	none	non-3GCR	No	Random cKp

Isolate ID	Classification	Detected biomarkers	ST/KL	Kleborate virulence score	CTX-M gene	AMR phenotype	Abscess complication	WGS selection category
KML26274	cKp	none	ST1966/KL1	0	none	non-3GCR	No	Random cKp
KML26275	cKp	none	ST17/KL2	0	none	non-3GCR	No	Random cKp
KML26276	hvKp	all 5	ST23/KL1	5	none	non-3GCR	Yes	hvKp
KML26279	cKp	none	ST1322/KL139	0	none	non-3GCR	No	Random cKp
KML26280	cKp	none	ST730/KL51	0	none	non-3GCR	No	Random cKp
KML26284	cKp	none	ST2055/KL14	0	none	non-3GCR	No	Random cKp
KML26285	cKp	*rmpA*, *iroB*, *peg-344*	ST39/KL23	0	none	non-3GCR	Yes	Added cKp
KML26286	cKp	none	ST353/KL110	1	*bla*$_{CTX-M}$ family	3GCR	No	Added cKp
KML26287	cKp	none	ST45/KL24	1	none	non-3GCR	No	Random cKp

Isolate ID	Classification	Detected biomarkers	ST/KL	Kleborate virulence score	CTX-M gene	AMR phenotype	Abscess complication	WGS selection category
KML26289	cKp	none	ST147/KL31	0	none	non-3GCR	No	Random cKp
KML26290	cKp	none	ST2123/KL28	0	none	non-3GCR	No	Random cKp
KML26291	cKp	none	ST353/KL110	1	*bla*$_{CTX-M-15}$	3GCR	No	Random cKp

ESBL production and 3GCR were defined phenotypically. CTX-M gene indicates the CTX-M β-lactamase gene detected by AMRFinderPlus. WGS selection category indicates whether the isolate was an hvKp isolate, a randomly sampled cKp isolate, or an additionally included cKp isolate selected to ensure representation of 1–4-biomarker or 3GCR isolates. Abbreviations: 3GCR, third-generation cephalosporin-resistant; AMR, antimicrobial resistance; cKp, classical *K. pneumoniae*; hvKp, hypervirulent *K. pneumoniae*; KL, capsule synthesis locus; ST, sequence type. All 5 indicates detection of *rmpA*, *rmpA2*, *iucA*, *iroB*, and *peg-344*.

Appendix Table 13. Sequence Type Distribution Among Sequenced Hypervirulent *Klebsiella pneumoniae*, Randomly Sampled Classical *K. pneumoniae*, and Third-Generation Cephalosporin-Resistant Isolates

		randomly sampled	
ST	hvKp (n = 28)	cKp (n = 125)	3GCR (n = 31)
23	10 (36)	1 (0.8)	0 (0.0)
65	8 (29)	0 (0.0)	0 (0.0)
412	4 (14)	0 (0.0)	2 (7)
86	2 (7)	0 (0.0)	0 (0.0)
268	1 (4)	1 (0.8)	0 (0.0)
29	1 (4)	0 (0.0)	0 (0.0)
893	1 (4)	0 (0.0)	1 (3)
37	0 (0.0)	10 (8)	7 (23)
45	0 (0.0)	9 (7)	2 (7)
22	0 (0.0)	5 (4)	0 (0.0)
200	0 (0.0)	5 (4)	0 (0.0)
307	0 (0.0)	5 (4)	8 (26)
353	0 (0.0)	4 (3)	7 (23)
Other	1 (4)	85 (68)	4 (13)

Data are n (%). Percentages are column percentages. Columns are not mutually exclusive; third-generation cephalosporin-resistant isolates may

		Randomly sampled	
ST	hvKp (n = 28)	cKp (n = 125)	3GCR (n = 31)

overlap with the hvKp and randomly sampled cKp columns. Abbreviations: 3GCR, third-generation cephalosporin-resistant; cKp, classical *K. pneumoniae*; hvKp, hypervirulent *K. pneumoniae*; ST, sequence type.

Appendix Table 14. Capsular Locus Distribution Among Sequenced Hypervirulent *Klebsiella pneumoniae*, Randomly Sampled Classical *K. pneumoniae*, and Third-Generation Cephalosporin-Resistant Isolates

		Randomly sampled	
KL	hvKp (n = 28)	cKp (n = 125)	3GCR (n = 31)
KL1	11 (39)	2 (2)	0 (0.0)
KL2	10 (36)	4 (3)	1 (3)
KL57	4 (14)	0 (0.0)	2 (7)
KL20	2 (7)	1 (0.8)	1 (3)
KL15	0 (0.0)	9 (7)	7 (23)
KL24	0 (0.0)	8 (6)	1 (3)
KL23	0 (0.0)	7 (6)	0 (0.0)
KL102	0 (0.0)	6 (5)	8 (26)
KL28	0 (0.0)	5 (4)	0 (0.0)

	Randomly sampled		
KL	hvKp (n = 28)	cKp (n = 125)	3GCR (n = 31)
KL110	0 (0.0)	4 (3)	7 (23)
Other	1 (4)	79 (63)	4 (13)

Data are n (%). Percentages are column percentages. Columns are not mutually exclusive; third-generation cephalosporin-resistant isolates may overlap with the hvKp and randomly sampled cKp columns. Abbreviations: 3GCR, third-generation cephalosporin-resistant; cKp, classical *K. pneumoniae*; hvKp, hypervirulent *K. pneumoniae*; KL, capsular locus.

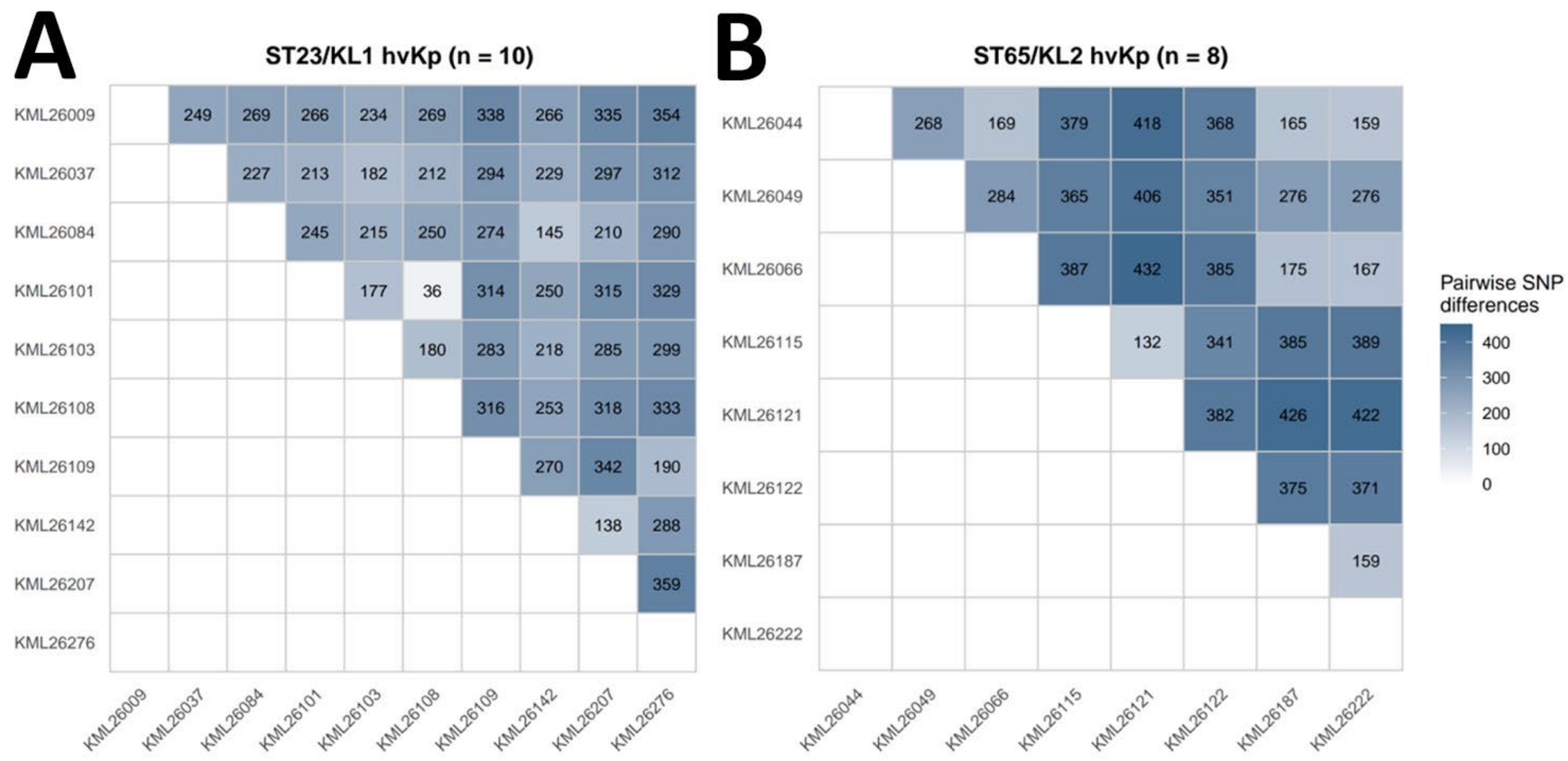

Appendix Figure. Pairwise Recombination-Filtered Core-Genome SNP Differences Among ST23/KL1 and ST65/KL2 Hypervirulent Klebsiella pneumoniae Isolates. Pairwise recombination-filtered core-genome SNP differences within predominant hvKp lineages. (A) ST23/KL1 hvKp isolates (n = 10). (B) ST65/KL2 hvKp isolates (n = 8). Values in the heatmap cells indicate pairwise SNP differences calculated from the recombination-filtered core-genome SNP alignment. Shading indicates pairwise SNP differences on a shared continuous scale across both panels; darker shading indicates larger SNP differences. Abbreviations: hvKp, hypervirulent K. pneumoniae; KL, capsular locus; ST, sequence type.
